# Fine-tuned deep transfer learning: an effective strategy for the accurate chronic kidney disease classification

**DOI:** 10.7717/peerj-cs.2800

**Published:** 2025-04-08

**Authors:** Zeshan Aslam Khan, Muhammad Waqar, Hashir Ullah Khan, Naveed Ishtiaq Chaudhary, Abeer TMA Khan, Iqra Ishtiaq, Farrukh Aslam Khan, Muhammad Asif Zahoor Raja

**Affiliations:** 1 Electrical and Computer Engineering, International Islamic University, Islamabad, Pakistan; 2 International Graduate Institute of Artificial Intelligence, National Yunlin University of Science and Technology, Yunlin, Taiwan; 3 Future Technology Research Center, National Yunlin University of Science and Technology, Yunlin, Taiwan; 4 Rawal Institute of Health Sciences, Rawalpindi, Pakistan; 5 Technological University Dublin, Dublin, Ireland; 6 Center of Excellence in Information Assurance, King Saud University, Riyadh, Saudi Arabia

**Keywords:** Transfer learning, Deep neural networks, Kidney disease, Disease diagnosis

## Abstract

Kidney diseases are becoming an alarming concern around the globe. Premature diagnosis of kidney disease can save precious human lives by taking preventive measures. Deep learning demonstrates a substantial performance in various medical disciplines. Numerous deep learning approaches are suggested in the literature for accurate chronic kidney disease classification by compromising on architectural complexity, classification speed, and resource constraints. In this study, deep transfer learning is exploited by incorporating unexplored yet effective variants of ConvNeXt and EfficientNetV2 for accurate and efficient classification of chronic kidney diseases. The benchmark computed tomography (CT)-based kidney database containing 12,446 CT scans of kidney tumor, stone cysts, and normal patients is utilized to train the designed fine-tuned networks. However, due to the highly imbalanced distribution of images among classes, the operation of data trimming is exploited for balancing the number of CT scans in each class, which is essential for designing an unbiased predictive network. By utilizing fine-tuned pre-trained models for our specific task, the training time is reduced leading to a computationally inexpensive solution. After the comprehensive hyperparameters tuning with respect to changes in learning rates, batch sizes, and optimizers, it is depicted that the designed fine-tuned EfficientNetV2B0 network of 23.8 MB in size with only 6.2 million architectural parameters shows substantial diagnostic performance by achieving a generalized test accuracy of 99.75% on balanced CT kidney database. Furthermore, the designed fine-tuned EfficientNetV2B0 attains high precision, recall, and F1-score of 99.75%, 99.63%, and 99.75%, respectively. Moreover, the final fine-tuned EfficientNetV2B0 ensures its scalability by achieving an impressive diagnostic accuracy of 99.73% on the test set of the original CT kidney dataset as well. Through the extensive evaluation of the proposed transfer learning strategy, it is concluded that the proposed design of fine-tuned EfficientNetV2B0 outperforms its counterparts in terms of accuracy and computational efficiency for chronic kidney disease diagnosis tasks. The final fine-tuned EfficientNetV2B0 serves as an accurate, efficient, and computationally inexpensive solution tailored for real-time deployment on medical or mobile edge devices.

## Introduction

Chronic kidney diseases (CKD) are becoming a major threat to the healthcare discipline due to their high mortality rates causing a large number of deaths. According to the study conducted on World Kidney Day in 2021, CKDs were accountable for almost 2.4 million deaths per year making CKD the 6th leading source of death (Kalantar-Zadeh et al., 2021). Artificial intelligence (AI) driven solutions have shown promising diagnostic outcomes in different healthcare applications (Cirrincione et al., 2023) by exploiting the enriching capabilities of supervised and unsupervised learning in the field of medical imaging. Supervised learning in medical imaging refers to the training of algorithms on annotated medical data (Papageorgiou, Dogoulis & Papageorgiou, 2023). The supervised learning approaches are successfully employed on various medical image analysis tasks involving MR/CT synthesis. Similarly, unsupervised learning (Barbiero et al., 2020) is another useful strategy in medical imaging where the algorithms do not rely on labeled data. Conversely, it identifies the latent feature characteristics within the provided data. The unsupervised learning (Kumari & Singh, 2024) approaches are useful for clustering in medical imaging where the labeled data may be unavailable.

The computed tomography (CT) and magnetic resonance imaging (MRI) (d’Anjou, 2024) are frequently used in imaging modalities both in modernized healthcare disciplines and AI-based solutions. MRI utilizes a powerful magnetic field and radio waves to generate an exhaustive representation of muscular tissues to detect structural imperfections in organs, muscles, cysts, and kidney morphologies. On the other hand, the CT imaging approach exploits X-rays to produce sharp-resolution tomographic scans for detailed visualization of bones and vascular and internal structures. CT scans are highly beneficial for detecting vascular abnormalities, internal hemorrhage, fractures, kidney stones or tumors, and kidney dilation or swelling. MRI and CT scans are widely used in AI-driven medical image analysis due to their detailed and interpretable visualization of human internal structures.

Recently, deep learning (DL) techniques (Petmezas et al., 2024) have been exploited in various image classification problems (JavadiMoghaddam, 2023; Tufail et al., 2024) due to their diverse capabilities for handling complex tasks. The ability of feature extraction and dimensionality reduction in various DL-based models make it a valuable approach for tackling different problems in image classification, such as disease diagnosis (Soni et al., 2024), object detection (Chen, Hei & Lai, 2023), anomaly detection (Khayyat, 2023), and intrusion detection (Soliman, Oudah & Aljuhani, 2023). DL-based image classification has noteworthy contributions in solving healthcare problems such as pneumonia disease (Ali et al., 2024), skin cancer disease (Thanh et al., 2020), oral cancer (Rahman et al., 2020), Alzheimer’s disease (Marwa et al., 2023), and chronic kidney diseases (Majid et al., 2023).

**Figure 1:**
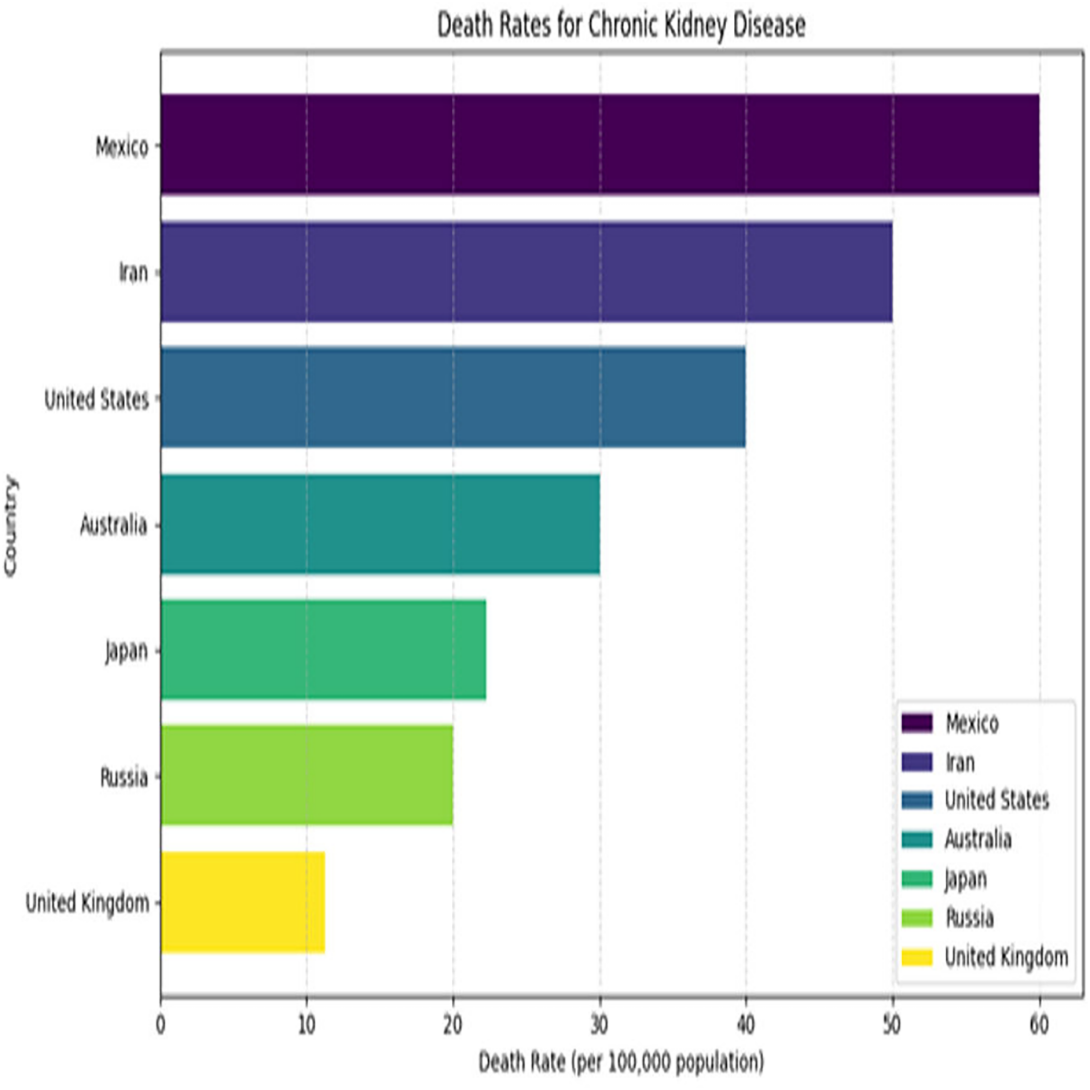
Death rates for kidney diseases.

**Figure 2:**
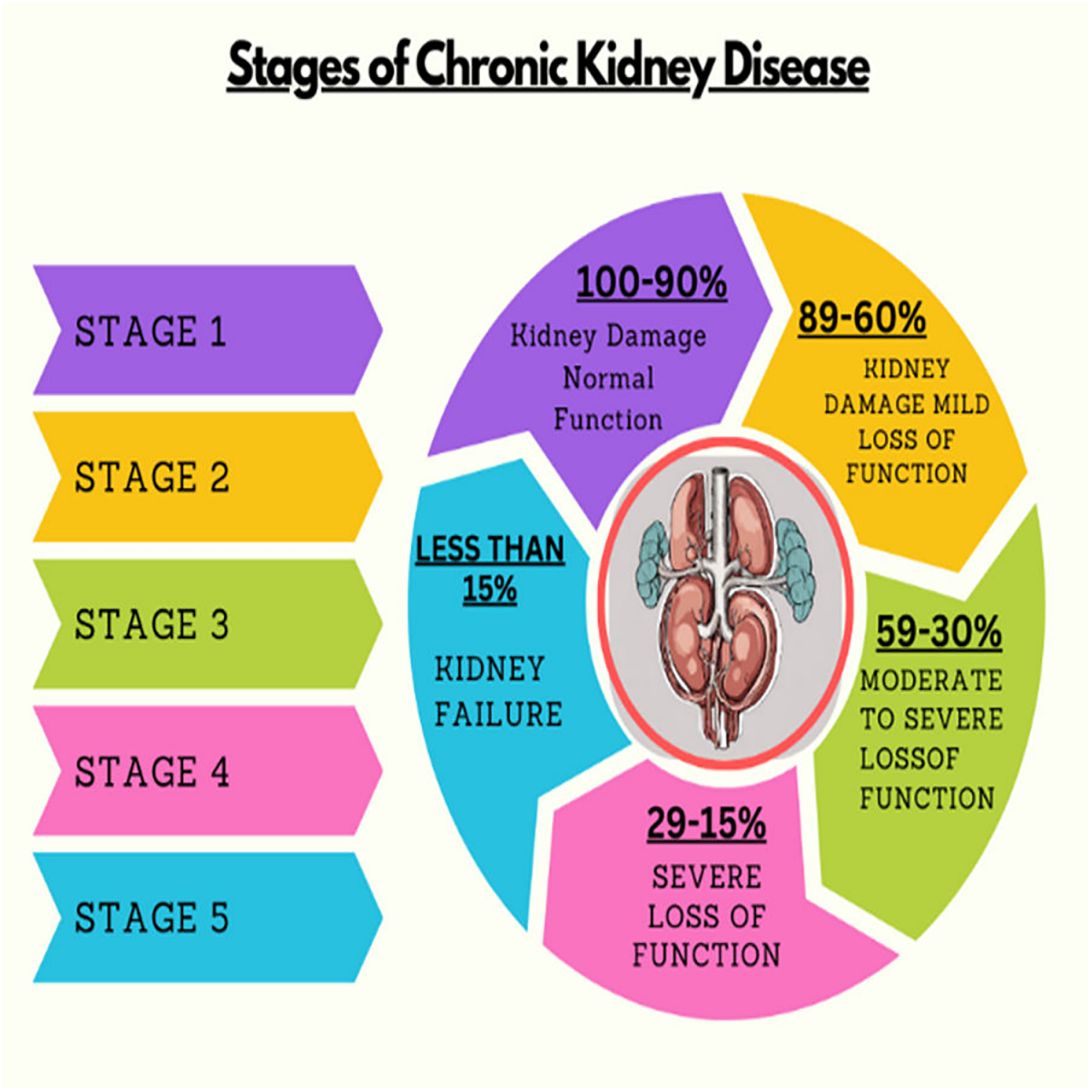
Stages of chronic kidney diseases.

Kidney plays a vital role in removing scum from blood. The rapid increase in kidney diseases is an alarming concern around the globe. A recent survey shows that approximately 10% of the global population suffers from chronic kidney diseases (Jha et al., 2013). In 2016, kidney disease was ranked as the 16th (Foreman et al., 2018) leading cause of death and it is expected to reach 5th by 2040. Figure 1 represents the number of deaths caused by chronic kidney disease in most affected regions of the world. Cysts, stones, and tumors are the most prevalent kidney diseases that can impair the smooth functionality of the kidney. Chronic kidney diseases lead to kidney failure through several stages, like kidney impairment, normal function, mild loss function, and moderate to severe loss function. The phases of chronic kidney diseases are graphically presented in Fig. 2.

Researchers have suggested numerous methods for the accurate and efficient classification of kidney diseases. Complex convolutional neural network (CNN)-based architectures utilized in existing methods, such as VGG16 and ResNet (Asif, Awais & Khan, 2023), result in computationally expensive models. To overcome this matter, the fine-tuned deep transfer learning-based unexplored yet effective models are exploited to save execution time by utilizing pre-trained architectures with respect to assigned tasks without compromising on the generalization capabilities. The probable limitations inferred from the existing works include efficiency, robustness, complex CNN architectures, accuracy, and computational expense.

This study exploits a transfer learning-based strategy by incorporating several EfficientNetV2 and ConvNeXt variants for accurate and efficient kidney disease classification. Existing benchmark architectures utilized in literature are complex, leading to computationally expensive solutions, so efficient and unexplored pre-trained models are incorporated to tackle the challenges of resource constraints. By utilizing pre-trained models, execution time is reduced as they do not require a training process from scratch and capitalize the previously learned knowledge. Therefore, this study utilizes the substantial capabilities of fine-tuned transfer learning to classify chronic kidney diseases accurately and effectively. The findings of the proposed work offer significant insights into clinical practices and disorders in computed tomography (CT). The key contributions of the proposed research are as follows:

- **Critical data pre-processing**: Data trimming is applied to the original CT kidney dataset to balance the distribution of CT scans among all classes, which is essential for avoiding predictive bias in the designed models.
- **Exploitation of transfer learning:** In this study, six pre-trained variants of EfficientNet & ConvNeXt namely ConvNeXtSmall, ConvNeXtTiny, EfficientNetV2M, EfficientNetV2B0, EfficientNetV2B1 and EfficientNetV2B3 are utilized, previously unexplored for kidney cancer diagnosis task.
- **Custom fine-tuning approach:** Designed a fine-tuned strategy in which the feature extractor of the pre-trained models is utilized as the backbone containing optimal ImageNet weights. Moreover, the suggested architectures are critically fine-tuned by adding task-specific classification layers.
- **Improved generalization & stability:** The incorporation of fine-tuned batch normalization and regularization techniques such as dropout, L1, and L2 regularization in the top layers of the architectures helps in improving the generalization and stability of the designed networks by tackling the overfitting scenarios efficiently.
- **Hyperparameter optimization:** Comprehensive hyperparameter tuning in terms of varying learning rates, batch sizes, and optimizers is performed to attain the optimal diagnostic performance.
- **Lightweight architectures:** The lightweight yet effective pre-trained networks are chosen to overcome the resource constraints without compromising on the diagnostic performance which will be beneficial for future deployment scenarios.
- **Extensive evaluation:** The comprehensive evaluation of the designed models is performed under distinct circumstances to achieve the best possible outcomes, providing valuable insights into the proposed approach over its counterparts.
- **Adaptability of transfer learning:** This research demonstrates the adaptability of the pre-trained networks mainly EfficientNet and ConvNeXt variants for a multi-class diagnostic task.

## Literature review

Numerous AI-driven approaches are presented for chronic kidney disease prediction through clinical metadata and CT imaging modality. Islam, Majumder & Hussein (2023) utilize 12 different machine learning (ML) algorithms for identifying chronic kidney disease through the clinical metadata comprised of 25 patient variables. Upon the training of the ML models, XGBoost shows the most prominent diagnostic performance by attaining a high accuracy of 0.983, precision, recall, and F1-score of 0.98. Similarly, Khan et al. (2020) explored seven machine learning algorithms such as naïve Bayes, logistic regression, support vector machine, composite hypercube on iterated random projection (CHIRP), decision tree, multi-layer perceptron and J48 for classifying the chronic kidney disease patients. The performance of the suggested algorithms was analyzed by monitoring the mean absolute error and accuracy, which shows that the CHIRP approach displays optimal classification performance by achieving high accuracy and low MAE of 99.75% and 0.0025. Furthermore, Rahman, Al-Amin & Hossain (2024) introduced an ensemble learning network comprised of 3 ML algorithms such as GDBT, XGBoost, and LightGBM. This approach shows substantial outcomes both in terms of achieving high accuracy and less computational cost. The suggested ensemble approach reaches a high accuracy, precision, recall, F1-score, and AUC-ROC of 99.75%, 99.40%, 99.41%, 99.61%, and 99.57%, respectively.

Similarly, a significant amount of DL-based strategies is suggested in the literature for the clinical analysis of chronic kidney diseases. Islam et al. (2022) present a study on comparative analysis of multiple approaches for chronic kidney disease classification on a standard CT kidney dataset. The six models, namely ResNet50, VGG16, InceptionV3, EANet, Swin Transformer, and CCT are incorporated in the study. The biggest challenge was the complex architecture of the models, which led to high computational resources. The best accuracy of 99.3% was attained by the Swin Transformer technique on the benchmark CT kidney database. Sundaramoorthy & Jayachandru (2023), exploit a pre-trained VGG16 model for the classification of kidney diseases. Due to the high architectural parameters of the VGG16 model, it takes a significant amount of time to train the model for the assigned task. An accuracy of 79% was achieved by the model and the solution was computationally expensive as well. Another study by Bhattacharjee et al. (2023), utilizes the capabilities of deep transfer learning by incorporating the Xception model for accurate and efficient classification among normal and affected CT scans. The suggested model shows non-linearity as no ReLU function was added within the architecture. With this approach, an accuracy of 99.3% was achieved for chronic kidney disease classification on the benchmark CT kidney dataset.

Researchers detect kidney disease by temporal electronic health results; for example, Ren et al. (2019), introduce a prognostic approach that analyzes patients by medical and lab records. Initially, the data is preprocessed by under-sampling and then fed to neural network and autoencoder. The projected method gains 67.9% classification accuracy. Krishnamurthy et al. (2021), suggest an approach that can detect the premature stage of kidney disease based on 3-, 6-, 12- and 24-month data records. They exploited several DL techniques (convolutional neural network, Bi-LSTM) and ML methods, including random forest, decision tree, and linear regression. The convolutional neural network achieved optimal results. On the other hand, Song et al. (2019) predict kidney disease by nontemporal electronic health records. Initially, the feature selection method is used to obtain features from electronic health records, and then for better accuracy, a deep neural network predictive variant is exploited. Bayram et al. (2022) present a detailed analysis of the performance of the YoloV7 model for kidney disease classification on US images database. The utilized database was very concise with only 658 CT scans. The low accuracy of 85% was attained by the suggested approach.

Badawy et al. (2023) suggest a fine-tuned deep transfer learning approach by utilizing six fine-tuned deep pre-trained models for the precise classification of chronic kidney diseases using a standard CT kidney dataset. A generalized accuracy of 98.2%, 98.8%, 99.4%, 99%, 94.8%, and 95.5% were attained through VGG16, VGG19, Xception, MobileNetv2, MobileNetv3, and NasNetMobile models. The groundbreaking limitations concluded by the study were resource constraints due to the excessive architectural parameters of these complex models. Badawy et al. (2023) recommended a lightweight CNN variant for accurate and efficient chronic kidney disease classification. No detailed description of the architectural layers is added to the study. An enhanced accuracy of 99.2% was achieved by the suggested approach on the CT kidney dataset. Yan & Razmjooy (2023), present a study on product-based solutions by exploiting deep belief network (DBN). The foremost challenge of the study was the hardware necessity for the deployment of DBN. With this strategy, a generalized accuracy of 97.8% was reached by the model on the CT kidney dataset.

The work by Ma et al. (2020), introduced a modified artificial neural network (ANN) variant for binary classification among normal and stone kidney CT scans. An improved accuracy of 97.5% was attained by the modified ANN approach. Sudharson & Kokil (2020), used an ensemble learning approach by combining three TL-based models namely ShuffleNet, MobileNetv2, and ResNet101 for correct chronic kidney disease classification. The 1,235 samples of CT scans for each cyst, normal, stone, and tumor class were utilized for training the model. A test accuracy of 96.5% was achieved by the suggested ensemble deep neural network technique. In the study by Aruna, Deepa & Devi (2023), a CNN-based variant of VGG19 was exploited for classifying the healthy and affected CT scans. The size of the VGG19 model is 500 MB with excessive architectural parameters, which takes enough time while training the model. Despite resource constraints, the suggested model achieves an optimal classification accuracy of 98% on the CT kidney database.

Another study by Mehedi et al. (2022), utilizes three different approaches, by influencing the abilities of heavy architectures like MobileNetV2, Inceptionv3, and VGG16 to achieve better generalized accuracy. An improved accuracy of 95.29%, 97.38%, and 99.48% was achieved by the respective models but due to large model sizes and training time, the solution was not computationally effective. Hossain et al. (2023) recommended a multi-feature fusion-based neural architecture strategy to enhance classification performance. The suggested approach mainly focuses on a better feature extraction mechanism to produce better results. With this strategy, an accuracy of 94.7% was achieved using the US images dataset. Qadir & Abd (2022) suggested an approach with two different models being utilized for assigned tasks. The study incorporates DenseNet201 architecture for feature extraction purposes and leverages the random forest model as a classifier instead of traditional dense layers of neural network for accurate and efficient chronic kidney disease classification. A benchmark accuracy of 99.44% was reached with the recommended approach. Pande & Agarwal (2024) utilize the You Only Look Once (YOLO) v8 network trained on the benchmark CT kidney dataset for chronic kidney disease classification. The suggested approach shows satisfactory performance by achieving accuracy, precision, recall, F1-score, and specificity of 82.52%, 85.76%, 75.28%, 75.72%, and 93.12%, respectively. Similarly, Sasikaladevi & Revathi (2024) introduced a hypergraph convolutional neural network (HCNN) in which hypergraph learning through graph convolutional networks (GCNs) is exploited for extracting the prominent features from CT scans. They achieved a substantial validation accuracy of 99.71% for chronic kidney disease diagnosis on the benchmark CT kidney dataset.

From the above detailed background, it is evident that various deep learning algorithms have shown satisfactory performance for chronic kidney disease diagnosis. However, the utilization of these AI-driven networks in real-world scenarios is limited or nearly unavailable due to the high computational cost of the models, which makes them unsuitable for deployment on resource-friendly medical or mobile devices. Furthermore, the strategy of fine-tuned transfer learning has been explored to a limited extent for classifying chronic kidney diseases through CT imaging modality, leaving a research gap for producing an accurate and computationally efficient solution for CKD diagnosis. Therefore, this study aims to exploit the capabilities of fine-tuned deep transfer learning for producing an accurate, computationally inexpensive, and easy-to-deploy network for filling the void between the capabilities of AI and practical deployment. Additionally, the proposed study intends to bridge the gap between accuracy and computational efficiency to produce a reliable solution for premature and timely diagnosis of CKDs.

## Materials AND methods

**Figure 3:**
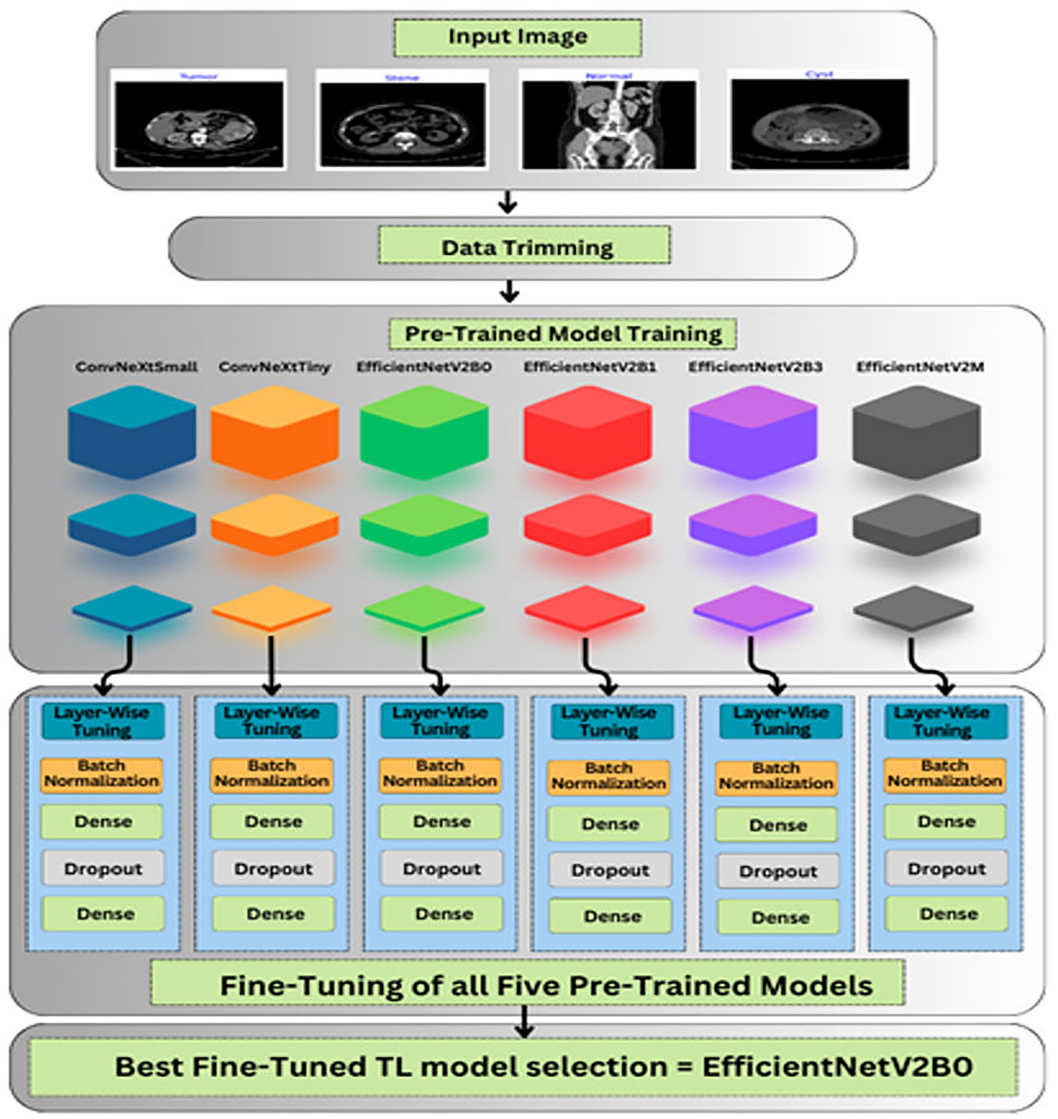
Graphical roadmap of the study.

The roadmap of the conducted research is explained in this section, which covers the description of databases, data preprocessing, and unexplored deep pre-trained models utilized to accurately and efficiently classify chronic kidney diseases. The graphical abstract of the research is represented in Fig. 3.

### Computing infrastructure

In this study, a kidney disease classification task was conducted on an HP VICTUS laptop equipped with a 12th-generation Intel Core i5 Octa-Core processor and 8 GB of RAM. The simulation work was executed using the Keras deep learning framework. These hardware and software specifications lead to efficient model training and evaluation.

### Database description

**Figure 4:**
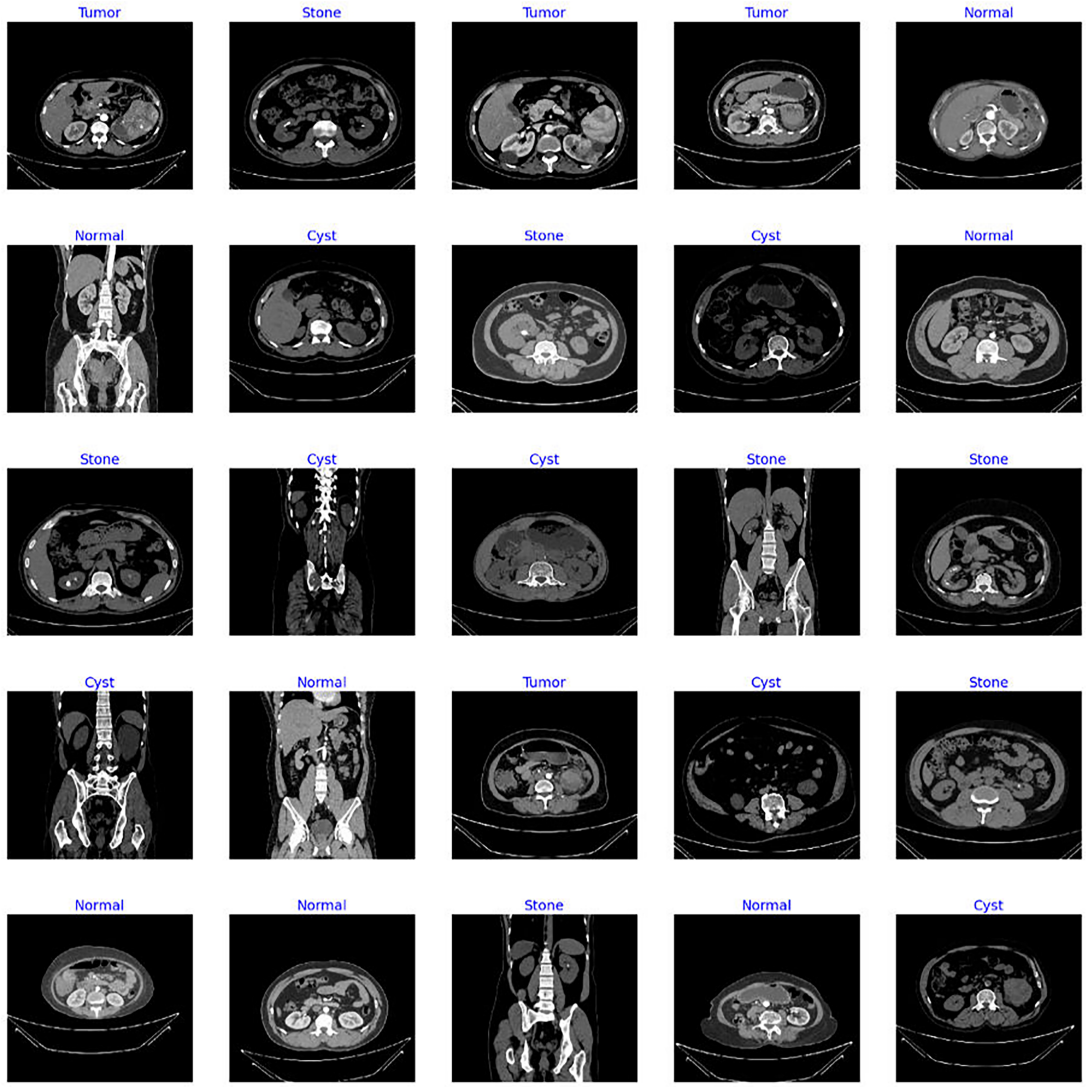
Sample images from standard CT kidney database.

The dataset utilized in this study is the “CT Kidney” dataset (Islam et al., 2022; https://www.kaggle.com/datasets/nazmul0087/ct-kidney-dataset-normal-cyst-tumor-and-stone) comprising 12,446 data samples with four classes of popular kidney diseases namely normal, cyst, stone, and kidney tumor. Figure 4 displays the sample images of each class from the standard CT kidney dataset.

The distribution of the CT scans for each class is highly imbalanced, containing 3,709 cyst scans, 2,283 kidney tumor scans, 1,377 kidney stone scans, and 5,077 scans of normal patients. Therefore, it is observed that the dataset is highly biased, with imbalanced distribution of samples among classes. Hence, the operation of data trimming is applied to the whole dataset where the dataset is divided into three sets of training, validation, and testing. The threshold set in the trimming process ensures that the training set includes 2,000 CT scans, with each class containing 500 CT scans. Similarly, 800 CT scans are reserved for the validation and testing sets, in which each class has an equal number of scans. The distribution of CT scans for each class in the original and processed dataset is shown in Table 1. The balancing of the images for each class is critical in order to produce an unbiased solution. The steps involved in the pre-processing of data are given in Table 2, which provides clear insights about the making of reliable processed data.

**Table 1:** Distribution of data samples in original & processed dataset with a split ratio of 70:15:15 for training, validation & testing.

Set	Labels	Original set	Processed (Trimmed) set
Training	Normal	3,554	500
	Cyst	2,596	500
	Tumor	1,598	500
	Stone	964	500
Validation	Normal	761	200
	Cyst	557	200
	Tumor	343	200
	Stone	206	200
Testing	Normal	762	200
	Cyst	556	200
	Tumor	242	200
	Stone	207	200

**Table 2:** Steps involved in data proprocessing.

Input: Database with 4 distinct classes Data Loading and statistical analysis. Due to imbalance dataset, data trimming is utilized to balance the number of samples, for each class in between maximum and minimum threshold of samples. ImageDataGenerator class is exploited to create training, validation and testing sets. Following parameters are specified for each set: Image Shape: (224, 250) Class Mode: ‘Categorical’ Color Space: ‘rgb’ Preprocessed data is provided to the proposed model.

### Deep transfer learning models

Transfer learning refers to the pre-trained models known for their diversity to solve complex tasks with ease by using prior learned information. The TL-based models are trained on a standard ImageNet (Krizhevsky, Sutskever & Hinton, 2017) dataset, comprised of a hundred thousand distinct classes. Fine-tuning these pre-trained models by adding own custom classifiers can aid to solve any classification problem. To save training and execution time, lightweight pre-trained models are exploited to tackle resource constraint issues without compromising the performance of the model. The six unexplored yet effective TL-based pre-trained models are utilized in this study for chronic kidney disease classification. Different variants of ConvNeXt (Liu et al., 2022) and EfficientNetV2 (Tan & Le, 2021) such as ConvNeXtSmall, ConvNeXtTiny, EfficientNetV2B0, EfficientNetV2B1, EfficientNetV2B3, EfficientNetV2M are exploited in this research. All chosen pre-trained models are cost-effective. A custom classifier is added instead of the top layers of TL-based models with specified count of classes.

### ConvNeXtTiny

ConvNeXtTiny (Khan & Hacı, 2023) is one of the pre-trained models, that belongs to the ConvNeXt family. The CNN-based ConvNet modules are included in its architecture and are known for their efficiency, scalability, and accuracy. The architecture exploits depth wise separable convolutional operations to overcome models’ complexity by keeping smaller variant sizes. This is a simplified yet effective pre-trained variant that contains 30 million architecture parameters.

### ConvNeXtSmall

Another CNN-based ConvNeXt model (Guzel et al., 2024) is primarily popular for its cost-effective computational performance. The model is designed to overcome resource constraints by evolving a compact model with reduced architecture size and parameters. The ConvNeXtSmall contains approximately 50 million parameters with a size of around 190 MBs.

### EfficientNetV2B0

This is a variant of the CNN-based EfficientNetV2 (Chougule et al., 2022) family, designed to attain high accuracy with low computational cost. EfficientNetV2B0 is a pre-trained variant with a simple architecture developed to bridge the gap between performance and model size and make it more suitable for classification tasks with limited computational resources.

### EfficientNetV2B1

EfficientNetV2B1 is another pre-trained (Marques, Agarwal & la Torre Díez, 2020) model, developed to improve the model efficiency by reducing the size of the architecture. This efficient variant contains only 7.2 million parameters. EfficientNetV2B1 model utilizes convolutional operations with a compound scaling strategy to enhance its effectiveness.

### EfficientNetV2B3

This is another variant (Liu et al., 2022) of the EfficientNetV2 series, designed for achieving high accuracy and efficiency in computer vision tasks. The architecture of the EfficientNetV2B3 model is more powerful and larger as compared to smaller variants like EfficientNetV2B1 and EffientNetV2V0. It is a little computationally expensive and contains more parameters but achieves higher quality results, particularly on complex datasets.

### EfficientNetV2M

It is a deep pre-trained model with medium-sized architecture where “M” in EfficientNetV2M (Saxena et al., 2023) stands for medium-sized variants in EfficientNetV2 series. It is designed to achieve a good balance between performance, model size, and computational cost. It provides better results on various computer vision tasks with fewer computational resources.

The characteristics of the chosen TL-based models incorporated in the research in terms of size and architectural parameters are represented in Table 3.

**Table 3:** Characteristics of TL-based models.

Model	Size (MB)	Parameters (million)
ConvNeXtSmall	190	50
ConvNeXtTiny	107	28
EfficientNetV2B0	23.8	6.2
EfficientNetV2B1	28	7.3
EfficientNetV2B3	50.8	13.3
EfficientNetV2M	204.3	53.4

### Custom fine-tuning of the TL-models

As shown in Fig. 3, after importing the feature extractor of the above TL-based models with their optimal pre-trained ImageNet weights, we have designed our custom classifier for the task at hand containing multiple batch normalization, dropout, and fully connected layers. In the feature extractor parts of the pre-trained models, the global max pooling is exploited, which is useful for prominent feature extraction and the formation of feature maps into a vector form before feeding the dense layers. Afterward, a batch normalization layer with momentum of 0.99 and epsilon of 0.001 is utilized for normalizing the values of feature maps from previous layers to enhance the training stability of the networks. Thereafter, a dense layer comprised of 256 units along with the L2 regularizer (*l* = 0.016), activity L1 regularizer (*l* = 0.006), and bias L1 regularizer (*l* = 0.006) is introduced to prevent overfitting scenarios. Additionally, a dropout layer with an optimal rate of 0.4 is utilized to avoid overfitting in the training process. At last, the final dense layer with 4 units along with SoftMax as an activation operator is exploited for a multi-class classification task at hand.

### Evaluation metrics

The performance of the proposed fine-tuned pre-trained networks for chronic disease classification is analyzed through the evaluation metrics (Rainio, Teuho & Klén, 2024) shown in Table 4, where T(+) and T(−) refer to the true positive and true negative, and F(+) and F(−) are false positive and false negative scenarios.

**Table 4:** Evaluation measures.

Accuracy (A)	$\displaystyle{{{T}\left( + \right) + {T}\left( - \right)} \over {{T}\left( + \right) + {T}\left( - \right) + {F}\left( - \right) + {F}\left( + \right)}}$
Precision (P)	$\displaystyle{{T\left( + \right)} \over {T\left( + \right) + F\left( + \right)}}$
Recall (R)	$\displaystyle{{T\left( + \right)} \over {T\left( + \right) + F\left( - \right)}}$
F1-Score (F1-S)	$2\; \times \displaystyle{{P\; \times R} \over {P + R}}$

## Results

In this study, six unexplored yet effective CNN-based pre-trained variants are utilized for the classification of chronic kidney diseases. The TL-based models are fine-tuned by leveraging the feature extraction portion and freezing the dense layer of the architecture. Furthermore, our own custom classifier comprises dropout, BatchNormalization, and fully connected layers with optimal hyperparameters added with a pre-trained base model for accurate, precise, and efficient chronic kidney disease diagnosis through CT scans. The custom callback of LR_ask is utilized in the study as well to monitor the loss and accuracy values during execution time and adjust the learning rate automatically with a specified rate, seeing the improvement in the loss term. All the above fine-tuned TL-based models are executed for 10 epochs initially, with a callback of the ask_epoch function, which provides insights about the learning behavior of the models after each five epochs and asks the users whether to continue or stop the training process by monitoring the convergence behavior of the networks. The result section of the study is divided into three sections, which play a critical role in fine-tuning the hyperparameters and obtaining the optimal ones for the best possible outcomes.

### Variations in learning rate

**Figure 5:**
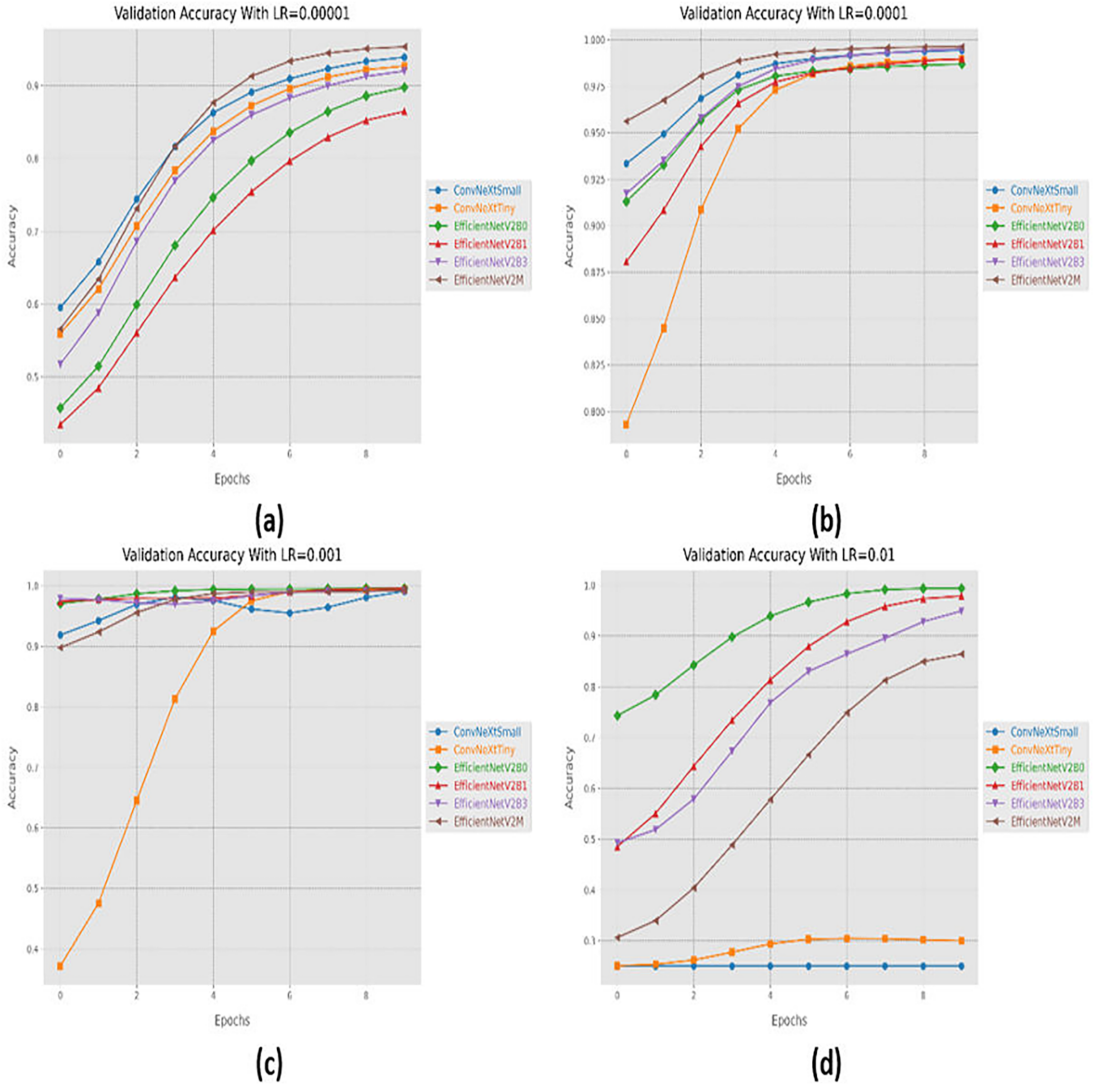
Convergence behavior in terms of accuracy for the suggested fine-tuned networks with respect to change in learning rates, (A) LR = 0.00001, (B) LR = 0.0001, (C) LR = 0.001, (D) LR = 0.01.

**Figure 6:**
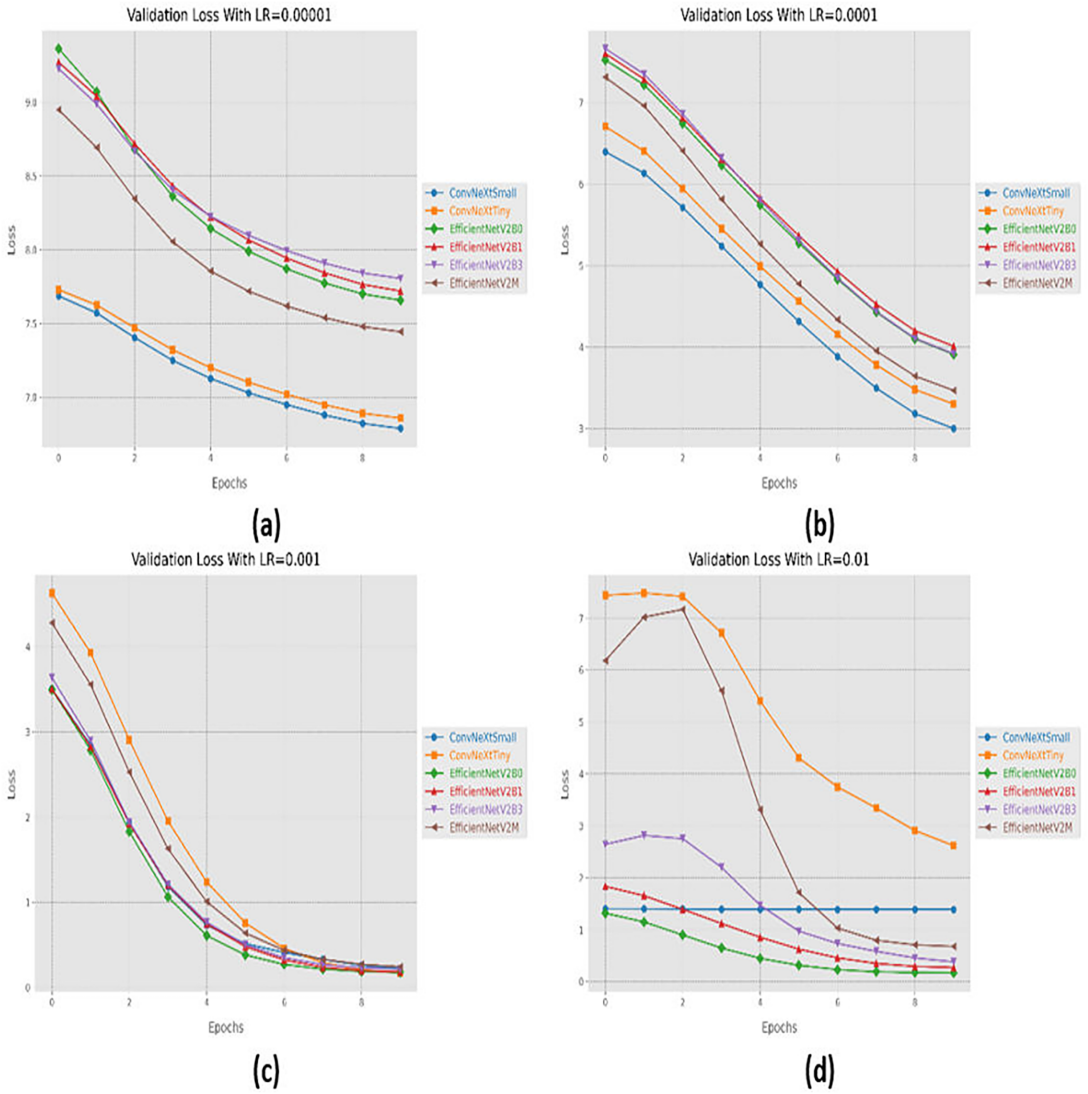
Convergence behavior in terms of loss for the proposed fine-tuned networks with respect to change in learning rates, (A) LR = 0.00001, (B) LR = 0.0001, (C) LR = 0.001, (D) LR = 0.01.

Initially, we started with four different learning rates in increasing order from 0.00001 to 0.01. The performance evaluation of the suggested networks, along with distinct learning rates is shown in Table 5. At first, all the fine-tuned pre-trained networks are executed with a learning rate of 0.00001, which displays that fine-tuned EfficinetNetV2M performs better as compared to its counterparts by achieving a good accuracy of 96%. Afterward, we gradually increased the learning rate from 0.00001 to 0.0001, which demonstrates significant improvement in the performance of all the designed architectures. With a learning rate of 0.0001, it is observed that fine-tuned ConvNeXtSmall shows remarkable classification performance by achieving a high accuracy of 99.38% with only 05 misclassified instances. Moreover, fine-tuned EfficientNetV2B0 and EfficientNetV2M with a learning rate of 0.0001 display the worst performance by attaining an accuracy of 98.88% as compared to its counterparts, which is far better with respect to the previous learning rate. The significant rise in the performance of the fine-tuned networks with higher learning rates further motivates us to test the higher learning rates in order to reach the optimal hyperparameters. Therefore, all the fine-tuned networks are executed with a learning rate of 0.001, which noteworthily enhances the performance of the designed fine-tuned architectures in which all the networks achieve an accuracy of more than 99% and fine-tuned EfficientNetV2B1 displays the best diagnostic performance by attaining a substantial accuracy of 99.50% with only 04 misclassified instances. Afterward, the learning rate is further increased from 0.001 to 0.01 for further performance analysis. However, it is depicted that with a learning rate of 0.01, the performances of the designed fine-tuned networks face a significant decline. With a learning rate of 0.01, fine-tuned EfficientNetV2B0 displays the best performance by reaching an accuracy of 99.25%. However, ConvNeXtSmall and ConvNeXtTiny show the worst performance by attaining low accuracies of 25% and 30.63%. From the variations in learning rate, we have shown that the performances of all the designed networks gradually increased with a higher learning rate, and the optimal learning rate for all the models was 0.001. Additionally, from Table 5, we have observed that the fine-tuned EfficientNetV2B0, EfficientNetV2B1, and EfficientNetV2M were among the best networks, performing remarkably for all variations of the learning rate. The learning and convergence behavior of the designed fine-tuned networks, along with distinct learning variations are graphically presented in Figs. 5A–5D and 6A–6D.

**Table 5:** Performance evaluation of the suggested fine-tuned networks with distinct learning rates.

Learning rate (LR)	Models	Execution time (h)	Time per epoch (s)	Inference time (s)	Labels	Precision	Recall	F1-score	Mis-classified instances	Accuracy
0.00001	ConvNeXtSmall	0.27	100.3	17	Cyst	0.9192	0.9100	0.9146	59	92.63
					Normal	0.9681	0.9100	0.9381		
					Stone	0.8889	0.9600	0.9231		
					Tumor	0.9343	0.9250	0.9296		
	ConvNeXtTiny	0.18	66.4	14	Cyst	0.9368	0.8900	0.9128	61	92.37
					Normal	0.8972	0.9600	0.9275		
					Stone	0.9246	0.9200	0.9223		
					Tumor	0.9391	0.9250	0.9320		
	EfficientNetV2B0	0.14	52.4	16	Cyst	0.9448	0.8550	0.8976	80	90.02
					Normal	0.8785	0.9400	0.9082		
					Stone	0.9069	0.9250	0.9158		
					Tumor	0.8756	0.8800	0.8778		
	EfficientNetV2B1	0.16	57.8	16	Cyst	0.8510	0.8850	0.8676	113	85.88
					Normal	0.8333	0.9250	0.8768		
					Stone	0.8528	0.8400	0.8463		
					Tumor	0.9075	0.7850	0.8418		
	EfficientNetV2B3	0.16	59.5	12	Cyst	0.9353	0.9400	0.9377	65	91.87
					Normal	0.9150	0.9150	0.9150		
					Stone	0.8995	0.9400	0.9193		
					Tumor	0.9263	0.8800	0.9026		
	EfficientNetV2M	0.26	97.1	15	Cyst	0.9497	0.9450	0.9474	32	96.00
					Normal	0.9704	0.9850	0.9777		
					Stone	0.9356	0.9450	0.9403		
					Tumor	0.9847	0.9650	0.9747		
0.0001	ConvNeXtSmall	0.26	94.9	19	Cyst	0.9949	0.9850	0.9899	05	99.38
					Normal	1.0000	1.0000	1.0000		
					Stone	0.9851	0.9900	0.9875		
					Tumor	0.9950	1.0000	0.9975		
	ConvNeXtTiny	0.18	65.0	12	Cyst	0.9949	0.9700	0.9823	07	99.12
					Normal	1.0000	1.0000	1.0000		
					Stone	0.9900	0.9950	0.9925		
					Tumor	0.9804	1.0000	0.9901		
	EfficientNetV2B0	0.13	49.9	07	Cyst	0.9802	0.9900	0.9851	09	98.88
					Normal	0.9851	0.9950	0.9900		
					Stone	0.9899	0.9850	0.9875		
					Tumor	1.0000	0.9850	0.9924		
	EfficientNetV2B1	0.12	45.5	16	Cyst	0.9949	0.9700	0.9823	08	99.00
					Normal	0.9950	1.0000	0.9975		
					Stone	0.9707	0.9950	0.9827		
					Tumor	1.0000	0.9950	0.9975		
	EfficientNetV2B3	0.15	55.2	12	Cyst	0.9900	0.9900	0.9900	06	99.25
					Normal	1.0000	1.0000	1.0000		
					Stone	0.9900	0.9900	0.9900		
					Tumor	1.0000	1.0000	1.0000		
	EfficientNetV2M	0.24	88.7	18	Cyst	0.9948	0.9600	0.9771	09	98.88
					Normal	1.0000	1.0000	1.0000		
					Stone	0.9660	0.9950	0.9803		
					Tumor	0.9950	1.0000	0.9975		
0.001	ConvNeXtSmall	0.29	107.1	27	Cyst	0.9801	0.9850	0.9825	07	99.12
					Normal	1.0000	1.0000	1.0000		
					Stone	0.9849	0.9800	0.9825		
					Tumor	1.0000	1.0000	1.0000		
	ConvNeXtTiny	0.16	60.8	14	Cyst	1.0000	0.9700	0.9848	07	99.12
					Normal	1.0000	0.9950	0.9975		
					Stone	0.9709	1.0000	0.9852		
					Tumor	0.9950	1.0000	0.9975		
	EfficientNetV2B0	0.12	43.9	08	Cyst	0.9949	0.9800	0.9874	06	99.25
					Normal	0.9950	1.0000	0.9975		
					Stone	0.9803	0.9950	0.9876		
					Tumor	1.0000	0.9950	0.9975		
	EfficientNetV2B1	0.15	56.0	17	Cyst	0.9850	0.9850	0.9850	04	99.50
					Normal	1.0000	1.0000	1.0000		
					Stone	0.9850	0.9850	0.9850		
					Tumor	1.0000	1.0000	1.0000		
	EfficientNetV2B3	0.21	76.4	12	Cyst	0.9850	0.9850	0.9850	07	99.12
					Normal	1.0000	0.9950	0.9975		
					Stone	0.9850	0.9850	0.9850		
					Tumor	0.9950	1.0000	0.9975		
	EfficientNetV2M	0.30	109.2	15	Cyst	0.9899	0.9800	0.9849	06	99.25
					Normal	1.0000	1.0000	1.0000		
					Stone	0.9802	0.9900	0.9851		
					Tumor	1.0000	1.0000	1.0000		
0.01	ConvNeXtSmall	0.29	107.0	24	Cyst	0.2500	1.0000	0.4000	600	25.00
					Normal	0.0000	0.0000	0.0000		
					Stone	0.0000	0.0000	0.0000		
					Tumor	0.0000	0.0000	0.0000		
	ConvNeXtTiny	0.17	62.6	18	Cyst	0.0000	0.0000	0.0000	555	30.63
					Normal	0.3119	0.9950	0.4749		
					Stone	0.2857	0.2300	0.2548		
					Tumor	0.0000	0.0000	0.0000		
	EfficientNetV2B0	0.08	29.7	11	Cyst	0.9900	0.9950	0.9925	06	99.25
					Normal	1.0000	0.9950	0.9975		
					Stone	0.9950	0.9900	0.9925		
					Tumor	0.9950	1.0000	0.9975		
	EfficientNetV2B1	0.09	35.6	10	Cyst	0.9894	0.9300	0.9588	27	96.63
					Normal	0.9848	0.9700	0.9773		
					Stone	0.9249	0.9850	0.9540		
					Tumor	0.9703	0.9800	0.9751		
	EfficientNetV2B3	0.12	45.3	12	Cyst	0.9648	0.9600	0.9624	31	96.13
					Normal	0.9510	0.9700	0.9604		
					Stone	0.9507	0.9650	0.9578		
					Tumor	0.9794	0.9500	0.9645		
	EfficientNetV2M	0.26	94.1	17	Cyst	0.9190	0.9650	0.9415	98	87.75
					Normal	0.7414	0.9750	0.8423		
					Stone	0.9767	0.8400	0.9032		
					Tumor	0.9419	0.7300	0.8225		

### Variations in batch size

**Figure 7:**
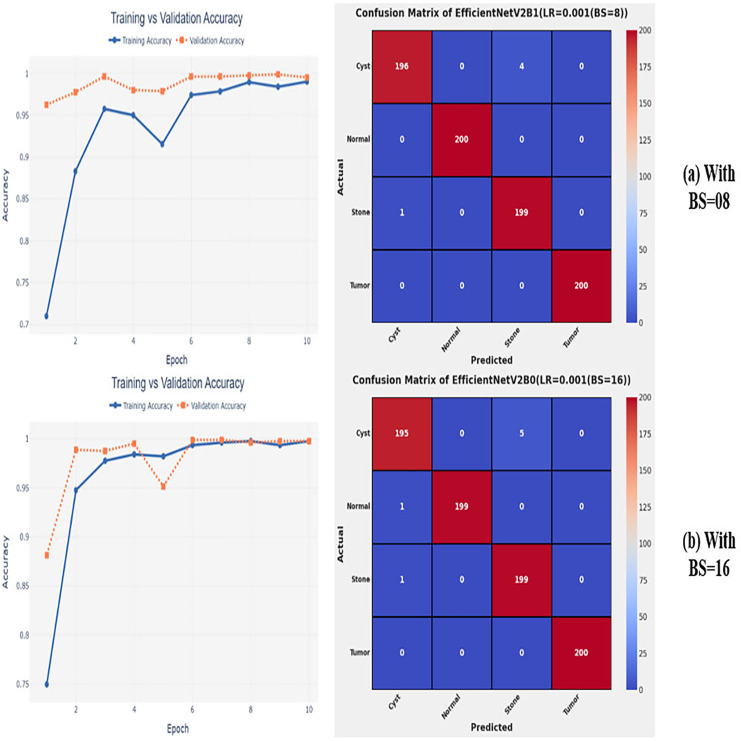
Convergence behavior & confusion matrix of the proposed fine-tuned networks with respect to change in batch sizes, (A) BS = 08, (B) BS = 16.

**Figure 8:**
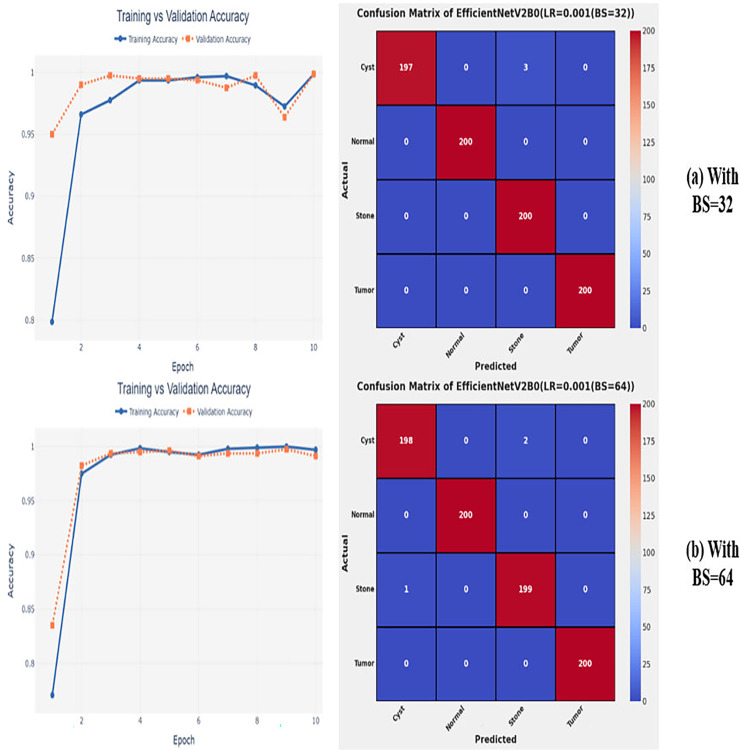
Convergence behavior & confusion matrix of the proposed fine-tuned networks with respect to change in batch sizes, (A) BS = 32, (B) BS = 64.

After the extensive performance evaluation of the fine-tuned networks with distinct learning rates, we have concluded that the most suitable learning rate is 0.001 for the task at hand. Thereafter, we executed the above best fine-tuned EfficientNetV2B0, EfficientNetV2B1, and EfficientNetV2M architectures with four different batch sizes along with an optimal learning rate of 0.001, computed earlier. The performance analysis of the models with variations in batch size along with optimal learning rate is given in Table 6. Initially, the networks are executed with a batch size of 8, in which fine-tuned EfficientNetV2B1 shows the best performance by achieving a generalized test accuracy of 99.38% with only 05 misclassified instances. Afterward, the models are executed with batch sizes of 8, 32, and 64, in which the fine-tuned EfficientNetV2B0 demonstrates the best performance among all by achieving a substantial accuracy of 99.12% and 99.62%, respectively. With the variations in batch sizes, we concluded that the fine-tuned EfficientNetV2B0 shows remarkable performance across all batch sizes. Figures 7A, 7B, and 8A, 8B display the overall performance evaluation of the networks on distinct batch sizes through the convergence curves and computed confusion matrix. From Table 6 it is depicted that the fine-tuned EfficientNetV2B0 is the best network among all in terms of its diagnostic performance with optimal learning rate and batch size of 0.001 and 32, respectively.

**Table 6:** Performance evaluation of the proposed fine-tuned networks with distinct batch sizes.

Batch size (BS)	Models	Execution time (hours)	Time per epoch (s)	Inference time (s)	Labels	Precision	Recall	F1-score	Mis-classified instances	Accuracy
8	EfficientNetV2B0	0.11	41.2	09	Cyst	0.9898	0.9700	0.9798	08	99.00
					Normal	1.0000	1.0000	1.0000		
					Stone	0.9706	0.9900	0.9802		
					Tumor	1.0000	1.0000	1.0000		
	EfficientNetV2B1	0.13	50.1	12	Cyst	0.9949	0.9800	0.9874	05	99.38
					Normal	1.0000	1.0000	1.0000		
					Stone	0.9803	0.9950	0.9876		
					Tumor	1.0000	1.0000	1.0000		
	EfficientNetV2M	0.34	125.3	20	Cyst	0.9949	0.9800	0.9874	05	99.38
					Normal	1.0000	1.0000	1.0000		
					Stone	0.9803	0.9950	0.9876		
					Tumor	1.0000	1.0000	1.0000		
16	EfficientNetV2B0	0.09	32.5	10	Cyst	0.9898	0.9750	0.9824	07	99.12
					Normal	1.0000	0.9950	0.9975		
					Stone	0.9755	0.9950	0.9851		
					Tumor	1.0000	1.0000	1.0000		
	EfficientNetV2B1	0.10	39.4	09	Cyst	1.0000	0.9650	0.9822	07	99.12
					Normal	1.0000	1.0000	1.0000		
					Stone	0.9662	1.0000	0.9828		
					Tumor	1.0000	1.0000	1.0000		
	EfficientNetV2M	0.29	106.0	21	Cyst	0.9804	1.0000	0.9901	10	98.75
					Normal	0.9849	0.9800	0.9825		
					Stone	1.0000	0.9850	0.9924		
					Tumor	0.9850	0.9850	0.9850		
32	EfficientNetV2B0	0.08	29.8	10	Cyst	1.0000	0.9850	0.9924	03	99.62
					Normal	1.0000	1.0000	1.0000		
					Stone	0.9852	1.0000	0.9926		
					Tumor	1.0000	1.0000	1.0000		
	EfficientNetV2B1	0.10	36.1	09	Cyst	0.9851	0.9950	0.9900	04	99.50
					Normal	1.0000	1.0000	1.0000		
					Stone	0.9949	0.9850	0.9899		
					Tumor	1.0000	1.0000	1.0000		
	EfficientNetV2M	0.26	96.5	20	Cyst	0.9852	1.0000	0.9926	03	99.62
					Normal	1.0000	1.0000	1.0000		
					Stone	1.0000	0.9850	0.9924		
					Tumor	1.0000	1.0000	1.0000		
64	EfficientNetV2B0	0.08	30.8	09	Cyst	0.9950	0.9900	0.9925	03	99.62
					Normal	1.000	1.0000	1.0000		
					Stone	0.9900	0.9950	0.9925		
					Tumor	1.0000	1.0000	1.0000		
	EfficientNetV2B1	0.09	34.1	09	Cyst	0.9898	0.9750	0.9824	09	98.88
					Normal	1.0000	0.9900	0.9950		
					Stone	0.9754	0.9900	0.9826		
					Tumor	0.9901	1.0000	0.9950		
	EfficientNetV2M	0.24	94.3	19	Cyst	0.9798	0.9800	0.9798	08	99.00
					Normal	1.0000	1.0000	1.0000		
					Stone	0.9806	0.9800	0.9902		
					Tumor	1.0000	1.0000	1.0000		

### Optimization variations

**Figure 9:**
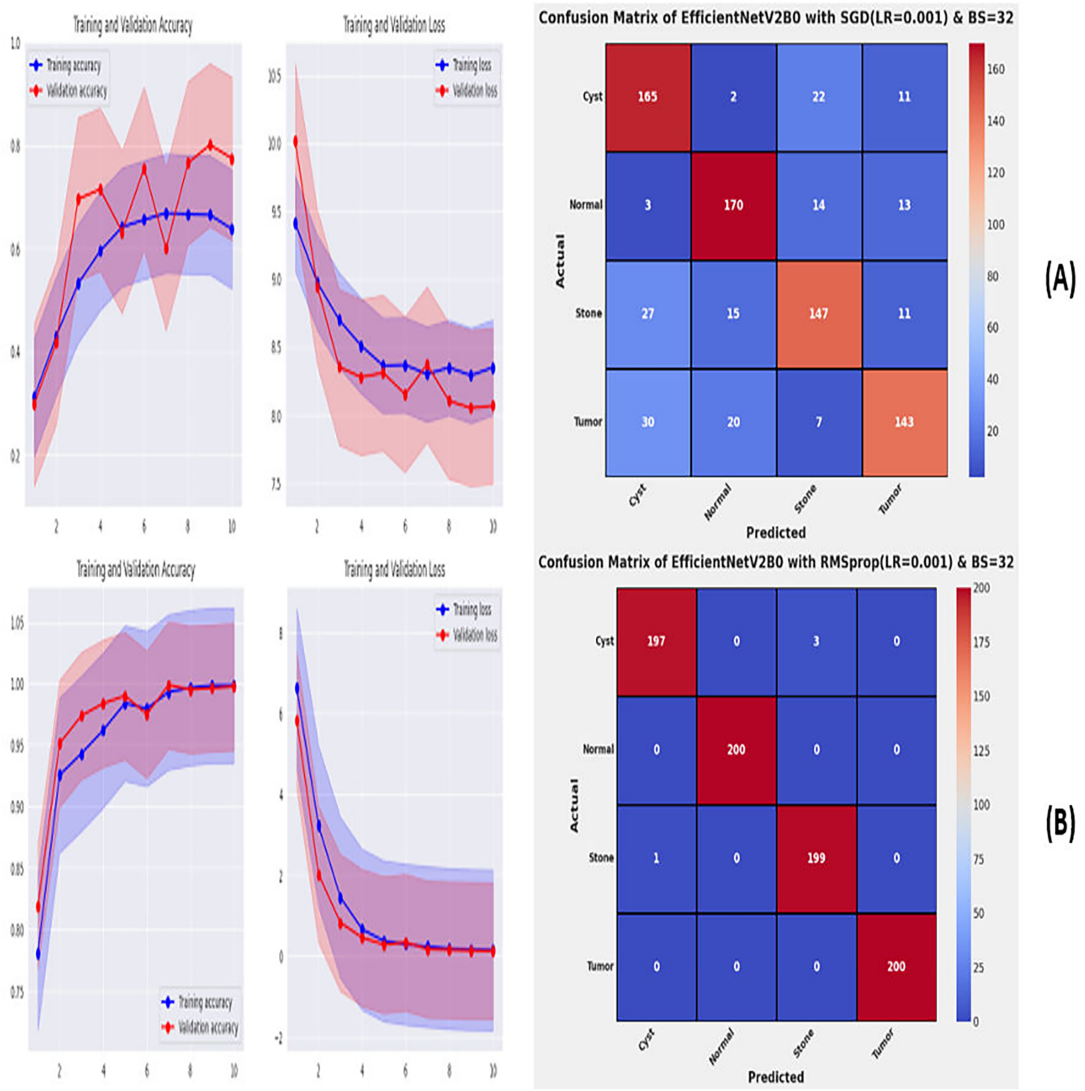
Convergence behavior & confusion matrix of the suggested fine-tuned networks with (A) SGD, (B) RMSPROP.

**Figure 10:**
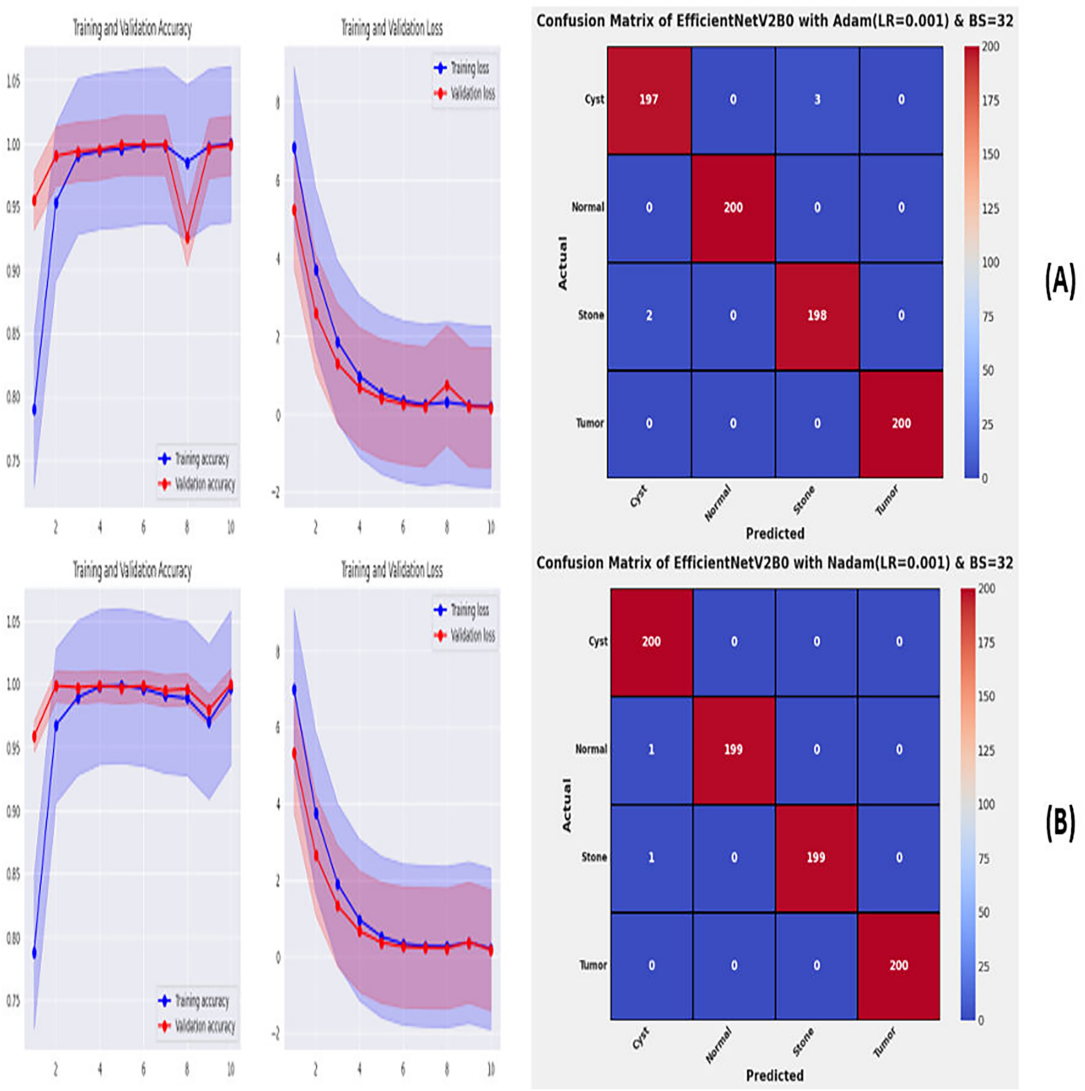
Convergence behavior & confusion matrix of the suggested fine-tuned networks with (A) ADAM, (B) NADAM.

From the comprehensive analysis of the performance of the models with respect to changes in learning rates and batch sizes, we have concluded that the fine-tuned EfficientNetV2B0, along with a learning rate of 0.001 and batch size of 32, shows the optimal performance. Thereafter, we executed the best model with optimal configurations along four different optimizers to seek further improvements in the classification performance. Table 7 presents the evaluation of the fine-tuned EfficientNetV2B0 with SGD, RMSprop, Adam, and Nadam optimizers. Figures 9A, 9B, and 10A, 10B show the effectiveness of the designed fine-tuned EfficientNetV2B0 with four distinct optimizers for chronic kidney disease diagnosis tasks through the convergence or learning curves and computed test confusion matrices. From Table 7, it is observed that the fine-tuned EfficientNetV2B0 shows the worst classification performance with SGD optimizer by attaining a low-test accuracy of 78.12%. However, with RMSprop and Adam optimizers, the suggested network displays satisfactory performance by reaching remarkable test accuracies of 99.50% and 99.38%. However, the fine-tuned EfficientNetV2B0 with a batch size of 32 and Nadam optimizers having an optimal learning rate of 0.001 achieves the best diagnostic accuracy of 99.75% with only 02 misclassified test instances.

**Table 7:** Performance evaluation of the proposed fine-tuned EfficientnetV2B0 with different optimization techniques.

Model	Optimizer	Execution time (h)	Time per epoch (s)	Inference time (s)	Labels	Precision	Recall	F1-Score	Mis-classified instances	Accuracy
EfficientNetV2B0	SGD	0.06	22.4	08	Cyst	0.7333	0.8250	0.7765	175	78.12
					Normal	0.8213	0.8500	0.8354		
					Stone	0.7737	0.7350	0.7538		
					Tumor	0.8034	0.7150	0.7566		
	RMSprop	0.06	22.5	09	Cyst	0.9949	0.9850	0.9899	04	99.50
					Normal	1.0000	1.0000	1.0000		
					Stone	0.9851	0.9950	0.9900		
					Tumor	1.0000	1.0000	1.0000		
	Adam	0.06	23.5	15	Cyst	0.9899	0.9850	0.9875	05	99.38
					Normal	1.0000	1.0000	1.0000		
					Stone	0.9851	0.9900	0.9875		
					Tumor	1.0000	1.0000	1.0000		
	Nadam	0.07	28.7	09	Cyst	0.9901	1.0000	0.9950	02	99.75
					Normal	1.0000	0.9950	0.9975		
					Stone	1.0000	0.9950	0.9975		
					Tumor	1.0000	1.0000	1.0000		

### Performance evaluation on original dataset for scalability analysis

**Figure 11:**
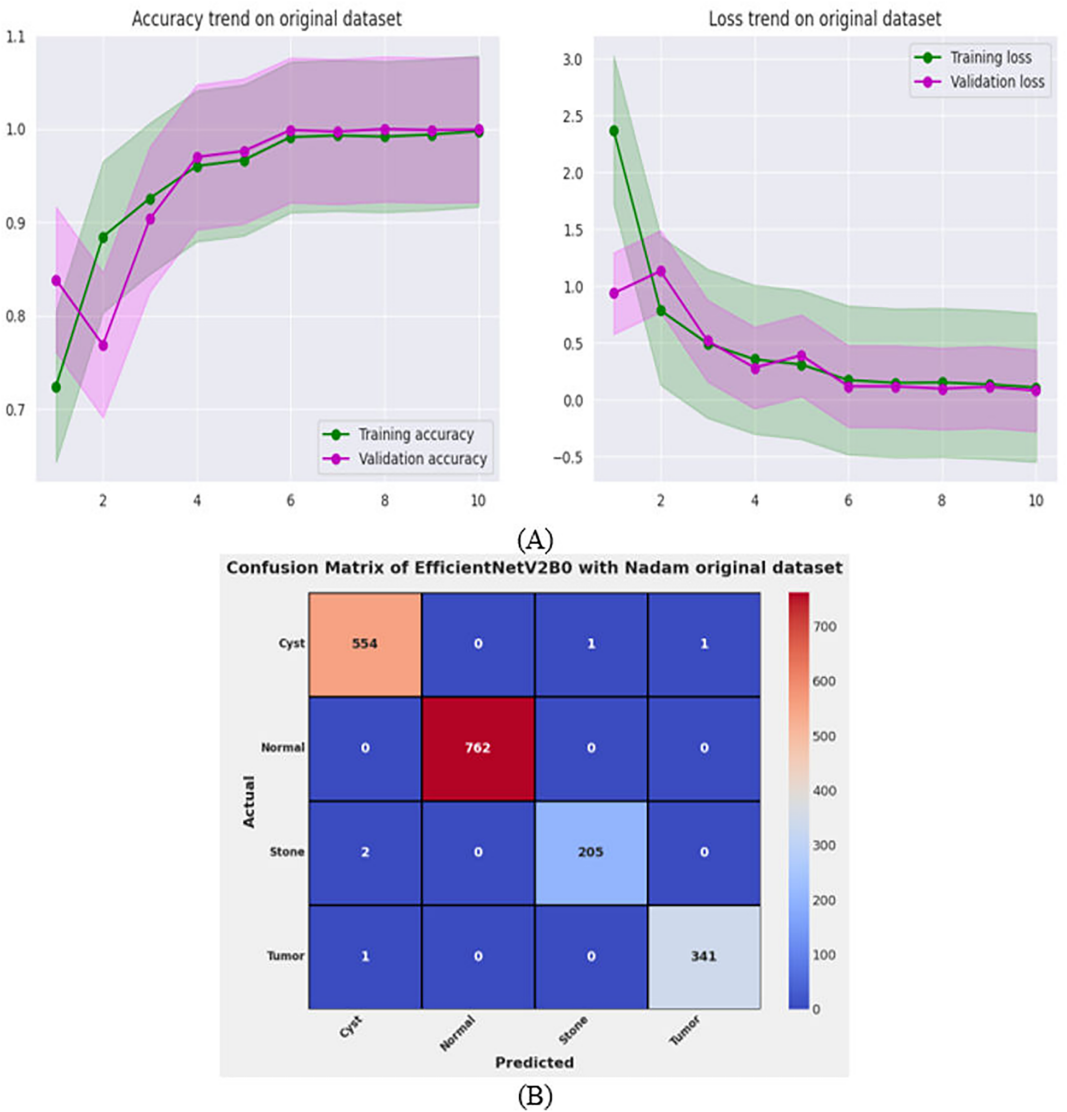
Convergence behavior & confusion matrix of the proposed fine-tuned Efficientnetv2b0 on original CT-kidney test dataset.

After the exhaustive experimental analysis of the fine-tuned TL-based networks on the balanced CT kidney dataset, it is depicted that the designed fine-tuned EfficientNetV2B0 with Nadam optimizer shows optimal diagnostic performance on a test set of a balanced CT kidney database. However, to validate the scalability of the proposed fine-tuned EfficientNetV2B0 network, we have executed the final architecture along with the optimal configuration of hyperparameters on the original CT kidney dataset with the split ratio of 70:15:15 for training, validation, and testing purposes, as shown in Table 8. The performance of the proposed fine-tuned EfficientNetV2B0 can be observed through the convergence curves and the computed predictive confusion matrix of the network on a test set of the original CT kidney dataset shown in Figs. 11A and 11B. From the learning or convergence curves, it is observed that there is a minimal gap between the training and validation accuracy, which is due to the varying distribution of images but it is not displaying any noteworthy overfitting. On the other hand, it is concluded that the proposed design of fine-tuned EfficientNetV2B0 is accurate and scalable as it shows substantial diagnostic performance on the test set of the original CT kidney database achieving an impressive classification accuracy of 99.73% with only 05 misclassified instances. However, the inference time of the network on the original CT kidney dataset is increased to 31 s, which is expected due to the large number of CT scans in the original set. At last, we conclude that the proposed fine-tuned EfficientNetV2B0 has emerged as an accurate, efficient, scalable, and computationally effective solution for chronic kidney disease classification tasks.

**Table 8:** Comparison of proposed fine-tuned EfficientnetV2B0 model with state-of-the-art (SOTA) models on benchmark CT kidney dataset.

Study	Dataset	Model	Model size (MB)	Architectural parameters (Millions)	Epoch	Accuracy(%)	Class	Precision	Recall	F1-score
Pande & Agarwal (2024)	CT Kidney dataset	YoloV8	98.8	25.9	50	82.52	Cyst	0.984	0.921	0.993
							Normal	0.749	0.100	0.769
							Stone	0.732	0.785	0.964
							Tumor	0.965	0.304	0.997
Sasikaladevi & Revathi (2024)	CT Kidney dataset	HCNN	-	-	200	99.71	Cyst	0.998	0.998	0.997
							Normal	0.996	0.100	0.997
							Stone	0.995	0.978	0.997
							Tumor	0.997	0.100	0.998
Mehedi et al. (2022)	CT Kidney dataset	MobileNet	13.37	3.5	120	95.29	Cyst	0.968	0.987	0.974
							Normal	0.992	0.951	0.952
							Stone	0.938	0.954	0.947
							Tumor	0.913	0.981	0.951
		VGG16	526.33	138	50	99.48	Cyst	0.975	0.96	0.983
							Normal	0.991	0.965	0.963
							Stone	0.972	0.951	0.961
							Tumor	0.981	0.964	0.995
		InceptionV3	91.16	23.9	68	97.38	Cyst	0.926	0.94	0.97
							Normal	0.963	0.84	0.974
							Stone	0.951	0.955	0.952
							Tumor	0.962	0.931	0.967
Islam et al. (2022)	CT Kidney dataset	EANET	251.77	66	100	77.02	Cyst	0.593	1	0.745
							Normal	0.876	0.848	0.871
							Stone	0.846	0.495	0.624
							Tumor	0.93	0.777	0.847
		Swin Transformer	190.73	50	100	99.30	Cyst	0.996	0.996	0.996
							Normal	0.996	0.981	0.988
							Stone	0.982	0.989	0.985
							Tumor	0.993	1	0.996
		CCT	76.29	20	100	96.54	Cyst	0.968	0.923	0.945
							Normal	0.989	0.975	0.982
							Stone	0.94	1	0.969
							Tumor	0.964	0.964	0.964
		VGG16	526.33	138	100	98.20	Cyst	0.996	0.968	0.982
							Normal	0.985	0.973	0.979
							Stone	0.966	0.988	0.977
							Tumor	0.983	0.996	0.989
		InceptionV3	91.16	23.9	100	61.60	Cyst	0.645	0.826	0.724
							Normal	0.584	0.898	0.708
							Stone	0.568	0.462	0.509
							Tumor	0.76	0.295	0.425
		ResNet-50	97.71	25.6	100	73.80	Cyst	0.735	0.641	0.685
							Normal	0.77	0.79	0.78
							Stone	0.745	0.692	0.717
							Tumor	0.706	0.827	0.762
Sudharson & Kokil (2020)	CT Kidney dataset	Ensemble DNN	188.20	49.4	_	96.54	Cyst	0.96	1	0.98
							Normal	0.94	0.98	0.96
							Stone	0.97	0.90	0.94
							Tumor	0.98	0.98	0.98
Qadir & Abd (2022)	CT Kidney dataset	DenseNet201 + Random Forest	76.67	20.1	_	99.44	Cyst	0.996	0.993	0.994
							Normal	0.989	1	0.994
							Stone	0.993	0.993	0.993
							Tumor	1	0.993	0.996
Fine-tined EfficientNetV2B0 (Proposed)	CT Kidney dataset	Transfer Learning	23.8	6.2	10	99.75	Cyst	0.9982	0.9982	0.9982
							Normal	1.0000	1.0000	1.0000
							Stone	0.9952	0.9952	0.9952
							Tumor	1.0000	1.0000	1.0000

## Discussion

In this study, fine-tuned deep TL-based models are exploited for the classification of chronic kidney diseases. The six unexplored yet effective deep fine-tuned pre-trained models are trained on a benchmark balanced CT kidney dataset. After comprehensive experimental analysis of all fine-tuned architectures with variations in learning rate, batch size, and optimizers, we have concluded that fine-tuned EfficientNetV2B0 along with batch size of 32 and Nadam optimizer with learning rate of 0.001 achieves a generalized test accuracy of 99.75% with only two misclassified instances on the test set of balanced CT kidney dataset. Extensive hyperparameter tuning is performed with respect to all possible variations to produce an optimal and reliable solution for accurate diagnosis of chronic kidney diseases. Furthermore, upon utilizing the original CT kidney dataset, it is observed that the designed fine-tuned EfficientNetV2B0 with optimal hyperparameters configuration shows impressive classification on the original dataset as well by attaining a substantial test accuracy of 99.73%, proving the scalability of the proposed strategy. The fine-tuned EfficientNetV2B0 attains improved evaluation metrics such as precision, recall, F1-score, and computational parameters as compared to other models on the benchmark CT kidney database. The proposed solution is computationally efficient due to its simplified architecture with only 6.2 million architectural parameters, which ensures that the final proposed fine-tuned EfficientNetV2B0 will serve as an accurate, efficient, and computationally inexpensive solution tailored for real-time deployment on medical or mobile edge devices.

### Comparison with benchmark models

**Figure 12:**
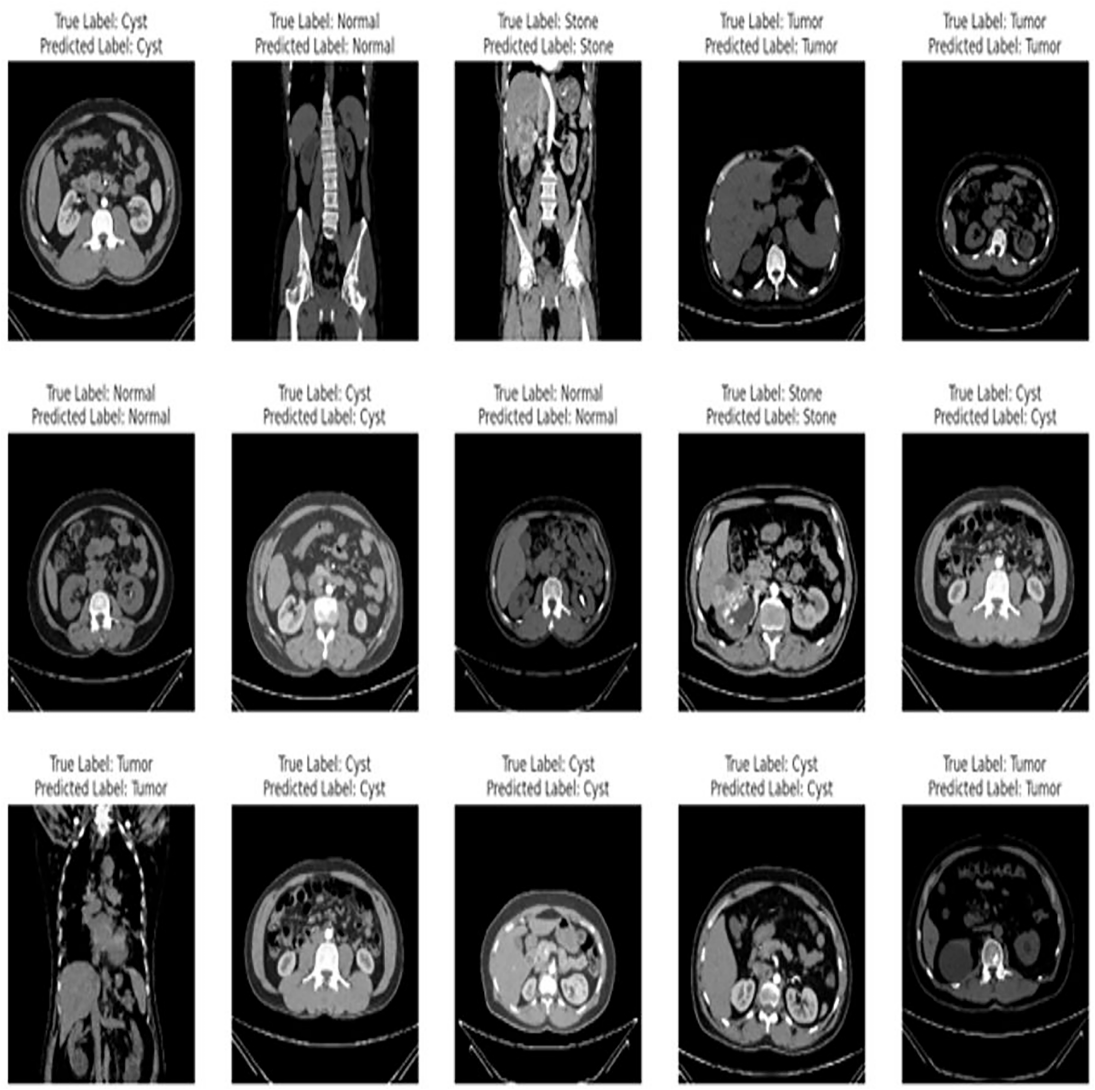
Generalized predictive capabilities of proposed fine-tuned EfficientnetV2B0 on unseen samples of benchmark CT kidney dataset.

The proposed fine-tuned EfficientNetV2B0 demonstrates vigorous predictive proficiencies by correctly and efficiently classifying various chronic kidney diseases. After an extensive training process on the benchmark CT kidney dataset, the fine-tuned EfficientNetV2B0 proves to be a generalized, scalable, and robust model for chronic kidney disease classification. Table 8 enlists the performance comparison of the suggested fine-tuned EfficientNetV2B0 model with existing state-of-the-art (SOTA) models on the benchmark CT kidney dataset. Notable improvements have been observed in terms of accuracy, precision, recall, and F1-score metrics by the proposed fine-tuned EfficientNetV2B0. After critical experimental analysis of Table 8, it is observed that the suggested approach outperformed existing benchmark models in terms of all specified evaluation metrics, which confirms the effectiveness of the proposed fine-tuned EfficientNetV2B0 in chronic kidney disease classification. Figure 12 demonstrates the generalization capabilities of the proposed fine-tuned EfficientNetV2B0 on unseen samples of the benchmark CT kidney dataset.

### Limitations and future directions

The proposed study mainly focuses on the latest TL-based CNN variants which are highly accurate and computationally inexpensive. However, the utilization of pre-trained transformers-driven architectures like Vision or Swin transformers and non-trivial feature extraction techniques may further enhance the diagnostic performance. Furthermore, the pre-trained variants are uninterpretable due to their black-box nature, which can be tackled by exploiting different techniques of explainable artificial intelligence (XAI) in the future for more understandable clinical decisions. Another probable limitation of the proposed research work is the utilization of a single dataset for the task at hand. However, this is due to the unavailability of any other benchmark database for the kidney disease classification task. However, for future perspective, the Generative AI-driven synthetic dataset can be developed to further validate the performance of the suggested transfer learning models for the analysis of kidney diseases.

## Conclusions

The study aimed to evaluate the performance of unexplored lightweight fine-tuned pre-trained variants for kidney disease classification. This study utilizes six different CNN-based fine-tuned TL models such as ConvNeXtTiny, ConvNeXtSmall, EfficientNetV2B0, EfficientNetV2B1, EfficientNetV2B3 and EfficientNetV2M on benchmark CT kidney dataset for kidney disease classification. The findings of the study are mentioned as follows:

- The selected TL-based variants are computationally cost-effective and can be executed with limited resources.
- It is depicted that quality performance can be achieved by utilizing transfer learning approaches for various computer vision tasks.
- The variations in hyperparameters like learning rate, batch size, and optimizers show noteworthy improvements in the performance of the suggested fine-tuned networks, which demonstrate the immense importance of hyperparameter tuning for achieving optimal results.
- The fine-tuned EfficientNetV2B0 with an optimal batch size of 32, initial leaning rate of 0.001, and Nadam optimizer displays remarkable classification performance by achieving generalized test accuracies of 99.75% and 99.73% on balanced and original CT kidney datasets ensuring the scalability of the proposed network.
- The proposed fine-tuned EfficientNetV2B0 network serves as an accurate, efficient, scalable, and computationally inexpensive solution for diagnosing kidney diseases tailored for real-time deployment on medical or mobile edge devices.

## Supplemental Information

10.7717/peerj-cs.2800/supp-1 Supplemental Information 1 Python code.

10.7717/peerj-cs.2800/supp-2 Supplemental Information 2 Raw data.

10.7717/peerj-cs.2800/supp-3 Supplemental Information 3 Readme.

10.7717/peerj-cs.2800/supp-4 Supplemental Information 4 Required packages to run the code files.

## Additional Information and Declarations

### Data Availability

The following information was supplied regarding data availability:

The code is available in the Supplemental File.

The CT Kidney Dataset is available at Kaggle: https://www.kaggle.com/datasets/nazmul0087/ct-kidney-dataset-normal-cyst-tumor-and-stone.

## References

[ref-1] Ali M, Shahroz M, Akram U, Mushtaq MF, Altamiranda SC, Obregon SA, Díez IDLT, Ashraf I (2024). Pneumonia detection using chest radiographs with Novel efficientNetV2L model. IEEE Access.

[ref-2] Aruna SK, Deepa N, Devi T (2023). A deep learning approach based on CT images for an automatic detection of polycystic kidney disease.

[ref-3] Asif S, Awais M, Khan SUR (2023). IR-CNN: inception residual network for detecting kidney abnormalities from CT images. Network Modeling Analysis in Health Informatics and Bioinformatics.

[ref-4] Badawy M, Almars AM, Balaha HM, Shehata M, Qaraad M, Elhosseini M (2023). A two-stage renal disease classification based on transfer learning with hyperparameters optimization. Frontiers in Medicine.

[ref-5] Badawy M, Almars AM, Balaha HM, Shehata M, Qaraad M, Elhosseini M, Bhandari M, Yogarajah P, Kavitha MS, Condell J (2023). A two-stage renal disease classification based on transfer learning with hyperparameters optimization, exploring the capabilities of a lightweight CNN model in accurately identifying renal abnormalities: cysts, stones, and tumors, using LIME and SHAP. Frontiers in Medicine.

[ref-6] Barbiero P, Lovino M, Siviero M, Ciravegna G, Randazzo V, Ficarra E, Cirrincione G (2020). Unsupervised multi-omic data fusion: the neural graph learning network.

[ref-7] Bayram AF, Gurkan C, Budak A, Karataş H (2022). A detection and prediction model based on deep learning assisted by explainable artificial intelligence for kidney diseases. European Journal of Science and Technology.

[ref-8] Bhattacharjee A, Rabea S, Bhattacharjee A, Elkaeed EB, Murugan R, Selim HMRM, Sahu RK, Shazly GA, Bekhit MMS (2023). A multi-class deep learning model for early lung cancer and chronic kidney disease detection using computed tomography images. Frontiers in Oncology.

[ref-9] Chen R, Hei L, Lai Yi (2023). Object detection in optical imaging of the Internet of Things based on deep learning. PeerJ Computer Science.

[ref-10] Chougule O, Katheria D, Jain K, Shinde S (2022). Tomato blight classification using transfer learning and fine tuning.

[ref-11] Cirrincione G, Cannata S, Cicceri G, Prinzi F, Currieri T, Lovino M, Militello C, Pasero E, Vitabile S (2023). Transformer-based approach to melanoma detection. Sensors.

[ref-12] d’Anjou M-A, Seiler G, Thrall DE (2024). Principles of computed tomography and magnetic resonance imaging. Thrall’s Textbook of Veterinary Diagnostic Radiology-E-Book.

[ref-13] Foreman KJ, Marquez N, Dolgert A, Fukutaki K, Fullman N, McGaughey M, Pletcher MA, Smith AE, Tang K, Yuan C-W, Brown JC, Friedman J, He J, Heuton KR, Holmberg M, Patel DJ, Reidy P, Carter A, Cercy K, Chapin A, Douwes-Schultz D, Frank T, Goettsch F, Liu PY, Nandakumar V, Reitsma MB, Reuter V, Sadat N, Sorensen RJD, Srinivasan V, Updike RL, York H, Lopez AD, Lozano R, Lim SS, Mokdad AH, Vollset SE, Murray CJL (2018). Forecasting life expectancy, years of life lost, and all-cause and cause-specific mortality for 250 causes of death: reference and alternative scenarios for 2016-40 for 195 countries and territories. The Lancet.

[ref-14] Guzel M, Kalkan M, Bostanci E, Acici K, Asuroglu T (2024). Cloud type classification using deep learning with cloud images. PeerJ Computer Science.

[ref-15] Hossain MS, Hassan SMN, Al-Amin M, Rahaman MN, Hossain R, Hossain MI (2023). Kidney disease detection from CT images using a customized CNN model and deep learning.

[ref-16] Islam MN, Hasan M, Hossain MK, Alam MGR, Uddin MZ, Soylu A (2022). Vision transformer and explainable transfer learning models for auto detection of kidney cyst, stone and tumor from CT-radiography. Scientific Reports.

[ref-17] Islam MA, Majumder MZH, Hussein MA (2023). Chronic kidney disease prediction based on machine learning algorithms. Journal of Pathology Informatics.

[ref-18] JavadiMoghaddam SM (2023). A novel framework based on deep learning for COVID-19 diagnosis from X-ray images. PeerJ Computer Science.

[ref-19] Jha V, Garcia-Garcia G, Iseki K, Li Z, Naicker S, Plattner B, Saran R, Wang AY-M, Yang C-W (2013). Chronic kidney disease: global dimension and perspectives. The Lancet.

[ref-20] Kalantar-Zadeh K, Li PK-T, Tantisattamo E, Kumaraswami L, Liakopoulos V, Lui S-F, Ulasi I, Andreoli S, Balducci A, Dupuis S, Harris T, Hradsky A, Knight R, Kumar S, Ng M, Poidevin A, Saadi G, Tong A (2021). World Kidney Day 2021: living well with kidney disease by patient and care partner empowerment—kidney health for everyone everywhere. American Journal of Kidney Diseases.

[ref-21] Khan T, Hacı İA (2023). Performance evaluation of enhanced ConvNeXtTiny-based fire detection system in real-world scenarios. https://openreview.net/forum?id=A-E41oZCfrf.

[ref-22] Khan B, Naseem R, Muhammad F, Abbas G, Kim S (2020). An empirical evaluation of machine learning techniques for chronic kidney disease prophecy. IEEE Access.

[ref-23] Khayyat MM (2023). Improved bacterial foraging optimization with deep learning based anomaly detection in smart cities. Alexandria Engineering Journal.

[ref-24] Krishnamurthy S, Ks K, Dovgan E, Luštrek M, Piletič BG, Srinivasan K, Li YC, Gradišek A, Syed-Abdul S (2021). Machine learning prediction models for chronic kidney disease using national health insurance claim data in Taiwan. Healthcare.

[ref-25] Krizhevsky A, Sutskever I, Hinton GE (2017). ImageNet classification with deep convolutional neural networks. Communications of the ACM.

[ref-26] Kumari S, Singh P (2024). Deep learning for unsupervised domain adaptation in medical imaging: recent advancements and future perspectives. Computers in Biology and Medicine.

[ref-27] Liu Z, Mao H, Wu C-Y, Feichtenhofer C, Darrell T, Xie S (2022). A convnet for the 2020s.

[ref-28] Liu D, Wang W, Wu X, Yang J (2022). EfficientNetv2 model for breast cancer histopathological image classification.

[ref-29] Ma F, Sun T, Liu L, Jing H (2020). Detection and diagnosis of chronic kidney disease using deep learning-based heterogeneous modified artificial neural network. Future Generation Computer Systems.

[ref-30] Majid M, Gulzar Y, Ayoub S, Khan F, Reegu FA, Mir MS, Jaziri W, Soomro AB (2023). Using ensemble learning and advanced data mining techniques to improve the diagnosis of chronic kidney disease. International Journal of Advanced Computer Science and Applications.

[ref-31] Marques G, Agarwal D, la Torre Díez ID (2020). Automated medical diagnosis of COVID-19 through EfficientNet convolutional neural network. Applied Soft Computing.

[ref-32] Marwa EL-G, Moustafa HEl-D, Khalifa F, Khater H, AbdElhalim E (2023). An MRI-based deep learning approach for accurate detection of Alzheimer’s disease. Alexandria Engineering Journal.

[ref-33] Mehedi MHK, Haque E, Radin SY, Rahman MAU, Reza MT, Alam MGR (2022). Kidney tumor segmentation and classification using deep neural network on CT images.

[ref-34] Pande SD, Agarwal R (2024). Multi-class kidney abnormalities detecting novel system through computed tomography. IEEE Access.

[ref-35] Papageorgiou VE, Dogoulis P, Papageorgiou D-P (2023). A convolutional neural network of low complexity for tumor anomaly detection.

[ref-36] Petmezas G, Papageorgiou VE, Vassilikos V, Pagourelias E, Tsaklidis G, Katsaggelos AK, Maglaveras N (2024). Recent advancements and applications of deep learning in heart failure: a systematic review. Computers in Biology and Medicine.

[ref-37] Qadir AM, Abd DF (2022). Kidney diseases classification using hybrid transfer-learning densenet201-based and random forest classifier. Kurdistan Journal of Applied Research.

[ref-38] Rahman MM, Al-Amin M, Hossain J (2024). Machine learning models for chronic kidney disease diagnosis and prediction. Biomedical Signal Processing and Control.

[ref-39] Rahman TY, Mahanta LB, Das AK, Sarma JD (2020). Histopathological imaging database for oral cancer analysis. Data in Brief.

[ref-40] Rainio O, Teuho J, Klén R (2024). Evaluation metrics and statistical tests for machine learning. Scientific Reports.

[ref-41] Ren Y, Fei H, Liang X, Ji D, Cheng M (2019). A hybrid neural network model for predicting kidney disease in hypertension patients based on electronic health records. BMC Medical Informatics and Decision Making.

[ref-42] Sasikaladevi N, Revathi A (2024). Digital twin of renal system with CT-radiography for the early diagnosis of chronic kidney diseases. Biomedical Signal Processing and Control.

[ref-43] Saxena A, Ajit A, Arora C, Raj G (2023). Efficient Net V2 algorithm-based NSFW content detection.

[ref-44] Soliman S, Oudah W, Aljuhani A (2023). Deep learning-based intrusion detection approach for securing industrial Internet of Things. Alexandria Engineering Journal.

[ref-45] Song X, Waitman LR, Hu Y, Yu ASL, Robins D, Liu M (2019). Robust clinical marker identification for diabetic kidney disease with ensemble feature selection. Journal of the American Medical Informatics Association.

[ref-46] Soni T, Gupta D, Uppal M, Juneja S, Gulzar Y, Ghafoor KZ (2024). Deep neural network framework for predicting cardiovascular diseases from ECG signals. Recent Advances in Computer Science and Communications.

[ref-47] Sudharson S, Kokil P (2020). An ensemble of deep neural networks for kidney ultrasound image classification. Computer Methods and Programs in Biomedicine.

[ref-48] Sundaramoorthy S, Jayachandru K (2023). Designing of enhanced deep neural network model for analysis and identification of kidney stone, cyst, and tumour. SN Computer Science.

[ref-49] Tan M, Le Q (2021). Efficientnetv2: Smaller models and faster training.

[ref-50] Thanh DNH, Prasath VBS, Hieu LM, Hien NN (2020). Melanoma skin cancer detection method based on adaptive principal curvature, colour normalisation and feature extraction with the ABCD rule. Journal of Digital Imaging.

[ref-51] Tufail H, Ahad A, Naqvi MH, Maqsood R, Pires IM (2024). Classification of vascular dementia on magnetic resonance imaging using deep learning architectures. Intelligent Systems with Applications.

[ref-52] Yan C, Razmjooy N (2023). Kidney stone detection using an optimized deep believe network by fractional coronavirus herd immunity optimizer. Biomedical Signal Processing and Control.

