# Supplementary material for: Fine-tuned deep transfer learning: an effective strategy for the accurate chronic kidney disease classification
Source: PeerJ Comput Sci. 2025 Apr 8;11:e2800. doi: 10.7717/peerj-cs.2800 (PMC12190387; doi:10.7717/peerj-cs.2800)
Supplement: Supplemental Information 3 [file peerj-cs-11-2800-s003.rtf]

Fine-tuned deep transfer learning: An effective strategy for the accurate chronic kidney disease classification
This research utilizes the enrich capabilities of deep transfer learning to accurately and efficiently diagnose different conditions of kidney diseases. The benchmark CT scan-based kidney dataset with four distinct class labels (Normal, Cyst, Tumor, and Stone) is used to train and validate the performance of the suggested models.
Dataset (URL/DOI):
CT Kidney Dataset: https://www.kaggle.com/datasets/nazmul0087/ct-kidney-dataset-normal-cyst-tumor-and-stone
Computing Infrastructure:
In this study, kidney disease classification task was conducted on a HP VICTUS laptop equipped with a 12th generation Intel Core i5 Octa-Core processor and 8 GBs of RAM. The simulation work was executed using the Keras deep learning framework. These hardware and software specifications lead to efficient model training and evaluation.

Preprocessing:
After completing the software requirements and dataset installation. The comprehensive preprocessing was performed on the dataset to make it compatible for the deep learning model as shown below:
STEPS INVOLVED IN DATA PROPROCESSING	
Input: Database with 4 distinct classes
·	Data Loading and statistical analysis.
·	Due to imbalance dataset, data trimming is utilized to balance the number of samples, for each class in between maximum and minimum threshold of samples.
·	Data augmentation technique is applied to reach the anticipated number of samples and for better generalization of the model.
·	Augmented dataframe is created to save the augmented samples with labels.
·	Balance function is defined to concatenate the original with augmented dataframe and return the balanced dataframe.
·	ImageDataGenerator class is exploited to create training, validation and testing sets.
·	Following parameters are specified for each set:
ü	Batch Size : 30
ü	Image Shape : (224, 250)
ü	Class Mode : 'Categorical'
ü	Color Space : 'rgb'
·	Preprocessed data is provided to the proposed model.	

Model Training:
The six unexplored yet effective transfer learning-based pre-trained models were utilized in this study for chronic kidney disease classification. Different variants of ConvNeXt and EfficientNetV2 such as ConvNeXtSmall, ConvNeXtTiny, EfficientNetV2B0, EfficientNetV2B1, EfficientNetV2B3, and EfficientNetV2M were exploited in this research. The description with respect to size and architectural parameters of all the models is already provided in the submitted manuscript, which confirms that the chosen pre-trained models are cost-effective.
Model Evaluation:
The performance of all the models is evaluated on test data through the following evaluation metrics:
·	Accuracy: To check how accurate (%) the models are for the kidney disease classification task: 

·	Classification Report: To evaluate the performance of the models in terms of precision (P), recall (R) and f1-score (F) for each class label for verify the biasness of the models, the classification reports are computed under all variations.
 ,
 ,

·	Confusion Matrix: To obtain the number of misclassified instances by the models on test dataset, confusion matrices are computed for all the models.
		
		
·	Inference Time: The execution time (hours) and time per epoch (second) are checked to compute the inference time of all the models, which gives valuable insights about the computational cost of the models.
Results:
The achieved accuracy, inference time, classification report, and confusion matrix for all the chosen models on test data is given and briefly discussed in the submitted manuscript. 
Limitations and Future Directions:
The only probable limitation of the proposed research work is the utilization of single dataset for the task at hand. But this is due to the unavailability of any other benchmark database for the kidney disease classification task. However, for future perspective the Generative Artificial Intelligence driven synthetic dataset can be developed to further validate the performance of the suggested transfer learning models for the analysis of kidney diseases. 

Implementation of the Project through Code Explanation:
The steps for understanding the implementation of the project are as follows:
·	Project structure
·	Installation
·	Overview of the functions used in preprocessing steps
·	Model architecture
·	Callbacks
·	Evaluating models performance
Project Structure:
o	Database: The Raw_Data.csv file provides the benchmark CT kidney dataset used in this study.
o	Code_python_files.zip: The main files containing preprocessing steps and models training code are provided in this zip file.
o	README file: The overall workflow of the proposed research with positive outcomes and concluded limitations is given in Updated Supplemental File (README).rtf.
o	Requirments: The necessary python packages and libraries used in this research are provided in the requirments.txt file.
Installation:
The necessary dependencies (libraries/ packages) associated with the kidney disease classification task were imported as follows:
import pandas as pd
import numpy as np
import os
import time
import matplotlib.pyplot as plt
import cv2
import seaborn as sns
import shutil
from sklearn.metrics import confusion_matrix, classification_report
from sklearn.model_selection import train_test_split
import tensorflow as tf
from tensorflow import keras
from tensorflow.keras.preprocessing.image import ImageDataGenerator
from tensorflow.keras.layers import Dense, Activation,Dropout,Conv2D, MaxPooling2D,BatchNormalization
from tensorflow.keras.optimizers import RMSprop, Nadam
from tensorflow.keras.applications import EfficientNetV2B1
from tensorflow.keras.metrics import categorical_crossentropy
from tensorflow.keras import regularizers
from tensorflow.keras.models import Model


Overview of the Functions Used in Preprocessing Steps
Various helper functions are utilized in preprocessing steps to make the input data compatible with the deep learning models. The description of the functions is given below:
o	make_dataframes(sdir): This function is use to load the data from source directory and further splits the data into three dataframes of training, validation and testing data for specified purposes.  The code of this helper function is given below:

def make_dataframes(sdir):
    filepaths=[]
    labels=[]
    classlist=sorted(os.listdir(sdir) )
    for klass in classlist:
        classpath=os.path.join(sdir, klass)
        if os.path.isdir(classpath):
            flist=sorted(os.listdir(classpath))
            desc=f'{klass:25s}'
            for f in tqdm(flist, ncols=130,desc=desc, unit='files', colour='blue'):
                fpath=os.path.join(classpath,f)
                filepaths.append(fpath)
                labels.append(klass)
    Fseries=pd.Series(filepaths, name='filepaths')
    Lseries=pd.Series(labels, name='labels')
    df=pd.concat([Fseries, Lseries], axis=1)
    train_df, dummy_df=train_test_split(df, train_size=.7, shuffle=True, random_state=123, stratify=df['labels'])
    valid_df, test_df=train_test_split(dummy_df, train_size=.5, shuffle=True, random_state=123, stratify=dummy_df['labels'])
    classes=sorted(train_df['labels'].unique())
    class_count=len(classes)
    sample_df=train_df.sample(n=50, replace=False)
    # calculate the average image height and with
    ht=0
    wt=0
    count=0
    for i in range(len(sample_df)):
        fpath=sample_df['filepaths'].iloc[i]
        try:
            img=cv2.imread(fpath)
            h=img.shape[0]
            w=img.shape[1]
            wt +=w
            ht +=h
            count +=1
        except:
            pass
    have=int(ht/count)
    wave=int(wt/count)
    aspect_ratio=have/wave
    print('number of classes in processed dataset= ', class_count)
    counts=list(train_df['labels'].value_counts())
    print(counts[0], type(counts[0]))
    print('the maximum files in any class in train_df is ', max(counts), '  the minimum files in any class in train_df is ', min(counts))
    print('train_df length: ', len(train_df), '  test_df length: ', len(test_df), '  valid_df length: ', len(valid_df))
    print('average image height= ', have, '  average image width= ', wave, ' aspect ratio h/w= ', aspect_ratio)
    return train_df, test_df, valid_df, classes, class_count

sdir=r'/content/CT-KIDNEY-DATASET-Normal-Cyst-Tumor-Stone/CT-KIDNEY-DATASET-Normal-Cyst-Tumor-Stone'
train_df, test_df, valid_df, classes, class_count=make_dataframes(sdir)

o	trim(df, max_samples, min_samples, column): Due to imbalance dataset, data trimming is utilized to balance the number of samples, for each class in between maximum and minimum threshold of samples.

def trim(df, max_samples, min_samples, column):
    df=df.copy()
    classes=df[column].unique()
    class_count=len(classes)
    length=len(df)
    print ('dataframe initially is of length ',length, ' with ', class_count, ' classes')
    groups=df.groupby(column)
    trimmed_df = pd.DataFrame(columns = df.columns)
    groups=df.groupby(column)
    for label in df[column].unique():
        group=groups.get_group(label)
        count=len(group)
        if count > max_samples:
            sampled_group=group.sample(n=max_samples, random_state=123,axis=0)
            trimmed_df=pd.concat([trimmed_df, sampled_group], axis=0)
        else:
            if count>=min_samples:
                sampled_group=group
                trimmed_df=pd.concat([trimmed_df, sampled_group], axis=0)
    print('after trimming, the maximum samples in any class is now ',max_samples, ' and the minimum samples in any class is ', min_samples)
    classes=trimmed_df[column].unique()# return this in case some classes have less than min_samples
    class_count=len(classes) # return this in case some classes have less than min_samples
    length=len(trimmed_df)
    print ('the trimmed dataframe now is of length ',length, ' with ', class_count, ' classes')
    return trimmed_df, classes, class_count

max_samples=500
min_samples=500
column='labels'
train_df, classes, class_count=trim(train_df, max_samples, min_samples, column)

o	balance(df, n, working_dir, img_size): Data augmentation techniques are applied to reach the anticipated number of samples and for better generalization of the model. 

def balance(df, n, working_dir, img_size):
    df=df.copy()
    print('Initial length of dataframe is ', len(df))
    aug_dir=os.path.join(working_dir, 'aug')# directory to store augmented images
    if os.path.isdir(aug_dir):# start with an empty directory
        shutil.rmtree(aug_dir)
    os.mkdir(aug_dir)
    for label in df['labels'].unique():
        dir_path=os.path.join(aug_dir,label)
        os.mkdir(dir_path) # make class directories within aug directory
    # create and store the augmented images
    total=0
    gen=ImageDataGenerator(horizontal_flip=True,  rotation_range=20, width_shift_range=.2,
                                  height_shift_range=.2, zoom_range=.2)
    groups=df.groupby('labels') # group by class
    for label in df['labels'].unique():  # for every class
        group=groups.get_group(label)  # a dataframe holding only rows with the specified label
        sample_count=len(group)   # determine how many samples there are in this class
        if sample_count< n: # if the class has less than target number of images
            aug_img_count=0
            delta=n - sample_count  # number of augmented images to create
            target_dir=os.path.join(aug_dir, label)  # define where to write the images
            msg='{0:40s} for class {1:^30s} creating {2:^5s} augmented images'.format(' ', label, str(delta))
            print(msg, '\r', end='') # prints over on the same line
            aug_gen=gen.flow_from_dataframe( group,  x_col='filepaths', y_col=None, target_size=img_size,
                                            class_mode=None, batch_size=1, shuffle=False,
                                            save_to_dir=target_dir, save_prefix='aug-', color_mode='rgb',
                                            save_format='jpg')
            while aug_img_count<delta:
                images=next(aug_gen)
                aug_img_count += len(images)
            total +=aug_img_count
    print('Total Augmented images created= ', total)
    # create aug_df and merge with train_df to create composite training set ndf
    aug_fpaths=[]
    aug_labels=[]
    classlist=os.listdir(aug_dir)
    for klass in classlist:
        classpath=os.path.join(aug_dir, klass)
        flist=os.listdir(classpath)
        for f in flist:
            fpath=os.path.join(classpath,f)
            aug_fpaths.append(fpath)
            aug_labels.append(klass)
    Fseries=pd.Series(aug_fpaths, name='filepaths')
    Lseries=pd.Series(aug_labels, name='labels')
    aug_df=pd.concat([Fseries, Lseries], axis=1)
    df=pd.concat([df,aug_df], axis=0).reset_index(drop=True)
    print('Length of augmented dataframe is now ', len(df))
    return df

·	make_gens(batch_size, train_df, test_df, valid_df, img_size): ImageDataGenerator class is exploited to create training, validation and testing sets. Following parameters are specified for each set:
ü	Batch Size : 30
ü	Image Shape : (224, 250)
ü	Class Mode : 'Categorical'
ü	Color Space : 'rgb'

def make_gens(batch_size, train_df, test_df, valid_df, img_size):
    trgen=ImageDataGenerator()
    t_and_v_gen=ImageDataGenerator()
    msg='{0:70s} for train generator'.format(' ')
    print(msg, '\r', end='') # prints over on the same line
    train_gen=trgen.flow_from_dataframe(train_df, x_col='filepaths', y_col='labels', target_size=img_size,
                                       class_mode='categorical', color_mode='rgb', shuffle=True, batch_size=batch_size)
    msg='{0:70s} for valid generator'.format(' ')
    print(msg, '\r', end='') # prints over on the same line
    valid_gen=t_and_v_gen.flow_from_dataframe(valid_df, x_col='filepaths', y_col='labels', target_size=img_size,
                                       class_mode='categorical', color_mode='rgb', shuffle=False, batch_size=batch_size)
    # for the test_gen we want to calculate the batch size and test steps such that batch_size X test_steps= number of samples in test set
    # this insures that we go through all the sample in the test set exactly once.
    length=len(test_df)
    test_batch_size=sorted([int(length/n) for n in range(1,length+1) if length % n ==0 and length/n<=80],reverse=True)[0]
    test_steps=int(length/test_batch_size)
    msg='{0:70s} for test generator'.format(' ')
    print(msg, '\r', end='') # prints over on the same line
    test_gen=t_and_v_gen.flow_from_dataframe(test_df, x_col='filepaths', y_col='labels', target_size=img_size,
                                       class_mode='categorical', color_mode='rgb', shuffle=False, batch_size=test_batch_size)
    # from the generator we can get information we will need later
    classes=list(train_gen.class_indices.keys())
    class_indices=list(train_gen.class_indices.values())
    class_count=len(classes)
    labels=test_gen.labels
    print ( 'test batch size: ' ,test_batch_size, '  test steps: ', test_steps, ' number of classes : ', class_count)
    return train_gen, test_gen, valid_gen, test_steps
batch_size=30
img_size=(224,250) # reduce image size to reduce training time at expense of model accuracy
train_gen, test_gen, valid_gen, test_steps =make_gens(batch_size, train_df, test_df, valid_df, img_size)

Network Architecture:
The six cost-effective and fine tuned pre-trained models are exploited in this research for chronic kidney disease classification task. The coding representation of the architectures are as follows:
from tensorflow.keras.applications import EfficientNetV2B0
from tensorflow.keras.optimizers import RMSprop
def make_model(img_size, lr):
    img_shape=(img_size[0], img_size[1], 3)
    base_model=EfficientNetV2B0(include_top=False, weights="imagenet",input_shape=img_shape, pooling='max')
    msg='Created ConvNeXtBase model'

    base_model.trainable=True
    x=base_model.output
    x=BatchNormalization(axis=-1, momentum=0.99, epsilon=0.001 )(x)
    x = Dense(256, kernel_regularizer = regularizers.l2(l = 0.016),activity_regularizer=regularizers.l1(0.006),
                    bias_regularizer=regularizers.l1(0.006) ,activation='relu')(x)
    x=Dropout(rate=.4, seed=123)(x)
    output=Dense(class_count, activation='softmax')(x)
    model=Model(inputs=base_model.input, outputs=output)
    model.compile(RMSprop(learning_rate=lr), loss='categorical_crossentropy', metrics=['accuracy', F1_score])
    msg=msg + f' with initial learning rate set to {lr}'
    print_in_color(msg)
    return model

lr=.001
model=make_model(img_size, lr) # using B3 model by default

Callbacks:
The customized LR_ask() function is explored as a callback to automatically adjusts the learning rate by monitoring the performance of the model during training which gives valuable insights on the models learning behavior. 
class LR_ASK(keras.callbacks.Callback):
    def __init__ (self, model, epochs,  ask_epoch, dwell=True, factor=.4): # initialization of the callback
        super(LR_ASK, self).__init__()
        self.model=model
        self.ask_epoch=ask_epoch
        self.epochs=epochs
        self.ask=True # if True query the user on a specified epoch
        self.lowest_vloss=np.inf
        self.lowest_aloss=np.inf
        self.best_weights=self.model.get_weights() # set best weights to model's initial weights
        self.best_epoch=1
        self.plist=[]
        self.alist=[]
        self.dwell= dwell
        self.factor=factor

    def get_list(self): # define a function to return the list of % validation change
        return self.plist, self.alist
    def on_train_begin(self, logs=None): # this runs on the beginning of training
        if self.ask_epoch == 0:
            print('you set ask_epoch = 0, ask_epoch will be set to 1', flush=True)
            self.ask_epoch=1
        if self.ask_epoch >= self.epochs: # you are running for epochs but ask_epoch>epochs
            print('ask_epoch >= epochs, will train for ', epochs, ' epochs', flush=True)
            self.ask=False # do not query the user
        if self.epochs == 1:
            self.ask=False # running only for 1 epoch so do not query user
        else:
            msg =f'Training will proceed until epoch {ask_epoch} then you will be asked to'
            print_in_color(msg )
            msg='enter H to halt training or enter an integer for how many more epochs to run then be asked again'
            print_in_color(msg)
            if self.dwell:
                msg='learning rate will be automatically adjusted during training'
                print_in_color(msg, (0,255,0))
        self.start_time= time.time() # set the time at which training started

    def on_train_end(self, logs=None):   # runs at the end of training
        msg=f'loading model with weights from epoch {self.best_epoch}'
        print_in_color(msg, (0,255,255))
        self.model.set_weights(self.best_weights) # set the weights of the model to the best weights
        tr_duration=time.time() - self.start_time   # determine how long the training cycle lasted
        hours = tr_duration // 3600
        minutes = (tr_duration - (hours * 3600)) // 60
        seconds = tr_duration - ((hours * 3600) + (minutes * 60))
        msg = f'training elapsed time was {str(hours)} hours, {minutes:4.1f} minutes, {seconds:4.2f} seconds)'
        print_in_color (msg) # print out training duration time

    def on_epoch_end(self, epoch, logs=None):  # method runs on the end of each epoch
        vloss=logs.get('val_loss')  # get the validation loss for this epoch
        aloss=logs.get('loss')
        if epoch >0:
            deltav = self.lowest_vloss- vloss
            pimprov=(deltav/self.lowest_vloss) * 100
            self.plist.append(pimprov)
            deltaa=self.lowest_aloss-aloss
            aimprov=(deltaa/self.lowest_aloss) * 100
            self.alist.append(aimprov)
        else:
            pimprov=0.0
            aimprov=0.0
        if vloss< self.lowest_vloss:
            self.lowest_vloss=vloss
            self.best_weights=self.model.get_weights() # set best weights to model's initial weights
            self.best_epoch=epoch + 1
            msg=f'\n validation loss of {vloss:7.4f} is {pimprov:7.4f} % below lowest loss, saving weights from epoch {str(epoch + 1):3s} as best weights'
            print_in_color(msg, (0,255,0)) # green foreground
        else: # validation loss increased
            pimprov=abs(pimprov)
            msg=f'\n validation loss of {vloss:7.4f} is {pimprov:7.4f} % above lowest loss of {self.lowest_vloss:7.4f} keeping weights from epoch {str(self.best_epoch)} as best weights'
            print_in_color(msg, (255,255,0)) # yellow foreground
            if self.dwell: # if dwell is True when the validation loss increases the learning rate is automatically reduced and model weights are set to best weights
                lr=float(tf.keras.backend.get_value(self.model.optimizer.lr)) # get the current learning rate
                new_lr=lr * self.factor
                msg=f'learning rate was automatically adjusted from {lr:8.6f} to {new_lr:8.6f}, model weights set to best weights'
                print_in_color(msg) # cyan foreground
                tf.keras.backend.set_value(self.model.optimizer.lr, new_lr) # set the learning rate in the optimizer
                self.model.set_weights(self.best_weights) # set the weights of the model to the best weights

        if aloss< self.lowest_aloss:
            self.lowest_aloss=aloss
        if self.ask: # are the conditions right to query the user?
            if epoch + 1 ==self.ask_epoch: # is this epoch the one for quering the user?
                msg='press enter to continue or enter a comment  below '
                print_in_color(msg)
                comment=input(' ')
                if comment !='':
                    print_in_color(comment, (155,245,66))
                msg='\n Enter H to end training or  an integer for the number of additional epochs to run then ask again'
                print_in_color(msg) # cyan foreground
                ans=input()

                if ans == 'H' or ans =='h' or ans == '0': # quit training for these conditions
                    msg=f'you entered {ans},  Training halted on epoch {epoch+1} due to user input\n'
                    print_in_color(msg)
                    self.model.stop_training = True # halt training
                else: # user wants to continue training
                    self.ask_epoch += int(ans)
                    if self.ask_epoch > self.epochs:
                        print('\nYou specified maximum epochs of as ', self.epochs, ' cannot train for ', self.ask_epoch, flush =True)
                    else:
                        msg=f'you entered {ans} Training will continue to epoch {self.ask_epoch}'
                        print_in_color(msg) # cyan foreground
                        if self.dwell==False:
                            lr=float(tf.keras.backend.get_value(self.model.optimizer.lr)) # get the current learning rate
                            msg=f'current LR is  {lr:8.6f}  hit enter to keep  this LR or enter a new LR'
                            print_in_color(msg) # cyan foreground
                            ans=input(' ')
                            if ans =='':
                                msg=f'keeping current LR of {lr:7.5f}'
                                print_in_color(msg) # cyan foreground
                            else:
                                new_lr=float(ans)
                                tf.keras.backend.set_value(self.model.optimizer.lr, new_lr) # set the learning rate in the optimizer
                                msg=f' changing LR to {ans}'
                                print_in_color(msg) # cyan foreground

Evaluating Models Performance:
The performance of the models is validated through their predictions on unseen test data. The confusion matrix and classification report are computed to visualize the number of correct and misclassified instances.
def predictor(test_gen):
    y_pred= []
    error_list=[]
    error_pred_list = []
    y_true=test_gen.labels
    classes=list(test_gen.class_indices.keys())
    class_count=len(classes)
    errors=0
    preds=model.predict(test_gen, verbose=1)
    tests=len(preds)
    for i, p in enumerate(preds):
        pred_index=np.argmax(p)
        true_index=test_gen.labels[i]  # labels are integer values
        if pred_index != true_index: # a misclassification has occurred
            errors=errors + 1
            file=test_gen.filenames[i]
            error_list.append(file)
            error_class=classes[pred_index]
            error_pred_list.append(error_class)
        y_pred.append(pred_index)

    acc=( 1-errors/tests) * 100
    msg=f'there were {errors} errors in {tests} tests for an accuracy of {acc:6.2f}'
    print_in_color(msg, (0,255,255), (100,100,100)) # cyan foreground
    ypred=np.array(y_pred)
    ytrue=np.array(y_true)
    f1score=f1_score(ytrue, ypred, average='weighted')* 100
    if class_count <=30:
        cm = confusion_matrix(ytrue, ypred )
        # plot the confusion matrix
        plt.figure(figsize=(12, 8))
        sns.heatmap(cm, annot=True, vmin=0, fmt='g', cmap='Blues', cbar=False)
        plt.xticks(np.arange(class_count)+.5, classes, rotation=90)
        plt.yticks(np.arange(class_count)+.5, classes, rotation=0)
        plt.xlabel("Predicted")
        plt.ylabel("Actual")
        plt.title("Confusion Matrix")
        plt.show()
    clr = classification_report(y_true, y_pred, target_names=classes, digits= 4) # create classification report
    print("Classification Report:\n----------------------\n", clr)
    return errors, tests, error_list, error_pred_list, f1score

errors, tests, error_list, error_pred_list, f1score =predictor(test_gen)
def print_errors(error_list):
    if len(error_list) == 0:
        print_in_color('There were no errors in predicting the test set')
    else:
        if len(error_list)<50:
            print ('Below is a list of test files that were miss classified \n')
            print ('{0:^30s}{1:^30s}'.format('Test File', ' Predicted as'))
            for i in range(len(error_list)):
                fpath=error_list[i]
                split=fpath.split('/')
                f=split[4]+ '-' + split[5]
                print(f'{f:^30s}{error_pred_list[i]:^30s}')
print_errors(error_list)
working_dir=r'./'
name='kidney-' + str(len(classes)) + '-(' + str(img_size[0]) + ' X ' + str(img_size[1]) + ')'
save_id=f'{name}-{f1score:5.2f}.h5'
model_save_loc=os.path.join(working_dir, save_id)
model.save(model_save_loc)
msg= f'model was saved as {model_save_loc}'
print_in_color(msg, (0,255,255), (100,100,100)) # cyan foreground
